# Integrated Omic Analysis Delineates Pathways Modulating Toxic TDP-43 Protein Aggregates in Amyotrophic Lateral Sclerosis

**DOI:** 10.3390/cells12091228

**Published:** 2023-04-24

**Authors:** Saiswaroop Rajaratnam, Akhil P. Soman, Kanikaram Sai Phalguna, Sai Sanwid Pradhan, Meghana Manjunath, Raksha Kanthavara Rao, Rajesh Babu Dandamudi, Sai Krishna Srimadh Bhagavatham, Sujith Kumar Pulukool, Sriram Rathnakumar, Sai Kocherlakota, Ashish Pargaonkar, Ravindra P. Veeranna, Natarajan Arumugam, Abdulrahman I. Almansour, Bibha Choudhary, Venketesh Sivaramakrishnan

**Affiliations:** 1 Disease Biology Lab, Department of Biosciences, Sri Sathya Sai Institute of Higher Learning, Prasanthi Nilayam, Anantapur 515134, Andhra Pradesh, India; 2 Central Water and Power Research Station, Khadakwasla, Pune 411024, Maharashtra, India; 3 Institute of Bioinformatics and Applied Biotechnology, Bengaluru 560100, Karnataka, India; 4 Phenomenex India, Hyderabad 500084, Telangana, India; 5 Laboratory of Cell Metabolism, Department of Pharmaceutical and Pharmacological Sciences, KU Leuven, 3000 Leuven, Belgium; 6 Application Division, Agilent Technologies Ltd., Bengaluru 560066, Karnataka, India; 7 Department of Biochemistry, Council of Scientific & Industrial Research (CSIR)-Central Food Technological Research Institute (CFTRI), Mysuru 570020, Karnataka, India; 8 Department of Chemistry, College of Science, King Saud University, P.O. Box 2455, Riyadh 11451, Saudi Arabia

**Keywords:** ALS, protein aggregates, neurodegenerative disease, transcriptomics, metabolomics

## Abstract

Amyotrophic lateral sclerosis (ALS) is a multi-systemic, incurable, amyloid disease affecting the motor neurons, resulting in the death of patients. The disease is either sporadic or familial with SOD1, C9orf72, FUS, and TDP-43 constituting the majority of familial ALS. Multi-omics studies on patients and model systems like mice and yeast have helped in understanding the association of various signaling and metabolic pathways with the disease. The yeast model system has played a pivotal role in elucidating the gene amyloid interactions. We carried out an integrated transcriptomic and metabolomic analysis of the TDP-43 expressing yeast model to elucidate deregulated pathways associated with the disease. The analysis shows the deregulation of the TCA cycle, single carbon metabolism, glutathione metabolism, and fatty acid metabolism. Transcriptomic analysis of GEO datasets of TDP-43 expressing motor neurons from mice models of ALS and ALS patients shows considerable overlap with experimental results. Furthermore, a yeast model was used to validate the obtained results using metabolite addition and gene knock-out experiments. Taken together, our result shows a potential role for the TCA cycle, cellular redox pathway, NAD metabolism, and fatty acid metabolism in disease. Supplementation of reduced glutathione, nicotinate, and the keto diet might help to manage the disease.

## 1. Introduction

Amyotrophic lateral sclerosis (ALS) is a complex multi-systemic disease associated with the loss of motor neurons in the brain stem, spinal cord, and motor cortex [1]. Neurodegenerative diseases like Amyotrophic Lateral Sclerosis, Alzheimer’s, Parkinson’s, and Huntington’s Disease are associated with protein aggregates that form amyloids [2]. The protein aggregates interfere with many important cellular functions eventually leading to neurodegeneration [3]. The disease is either sporadic or familial [4]. About 853 genes are associated with ALS, of which Single Nucleotide Polymorphisms (SNPs) in Superoxide Dismutase (SOD1), Chromosome-9 orf-72 (C9orf72), Fused in Sarcoma (FUS), and Tar DNA Binding Protein-43 (TDP-43) constitute the majority of familial ALS [5]. Multiple pathways are shown to be perturbed in the disease, including endoplasmic reticulum stress, proteasomal dysfunction, mitochondrial dysfunction, metabolic deregulation, oxidative stress, protein aggregation, and neuromuscular junction abnormalities [6]. These pathways could potentially modulate the course and outcome in ALS.

Transcriptomic, proteomic, and metabolomic analyses have been carried out on ALS patients and transgenic model systems with SNP in SOD1, C9orf72, FUS, and TDP-43 to understand the disease [7]. Further, the identified SNPs in TDP-43 show increased TDP-43 amyloids in patients with ALS [8]. Mutation of TDP-43 leads to aggregation in the cytoplasm and other ALS-related pathology.

The TDP-43 gene is located on chromosome number 18. The protein is made up of 414 amino acids [9]. Most of the mutations observed in TDP-43-induced ALS patients are missense mutations [10]. In TDP-43, two RNA Recognition Motifs: RRM1 and RRM2 have nuclear export signal sequences. The N-terminal region has nuclear localization signals. The C-terminal region has a prion-like domain and a glycine-rich region. TDP-43 is found to be a dimeric protein. Dimerization happens with the help of residues along the N-terminal region. This process is critical for RNA splicing [11]. Mutation in NLS sequences and RRM regions is found to cause cytoplasmic aggregation in yeast [12]. Mutations in exon 6 of the C-terminal region are found to be a major player in ALS pathology. Commonly observed mutations are A382T and M337V. Other mutations like A315T, Q331K, D169G, G294A/V, and Q343R are also well-studied [13]. Transcriptomic analysis of mice model of TDP-43 has shown a role in signaling pathways, inflammation, and metabolic pathways [14]. The metabolic pathways include the branch chain amino acid pathway, glycolysis, the TCA cycle, the electron transport chain, and fatty acid metabolism [15]. Consistent with this, mitochondrial dysfunction is observed in patients and model systems [16]. In addition, TDP-43 associated ALS displays mitochondrial fragmentation as well as alterations in fission and fusion [17]. Studies have also shown an association of epigenetic changes with the disease [18]. In TDP-43 models, an increased Acetyl CoA and an associated increased global acetylation of histones were observed [19]. Changes in the metabolic profile are a cumulative outcome of the changes in the genomic, transcriptomic, and proteomic profiles. Hence, metabolome is very near to the phenotype, and metabolic pathways could potentially be targeted for better outcomes in ALS. TDP-43 aggregation is shown to play a role in the pathophysiology of ALS [20]. Several mechanisms have been proposed for TDP-43 misfolding due to mutations and its role in ALS pathogenesis. Deregulation in TDP-43 protein clearance, impaired autophagy, inhibition of endocytosis, and mitochondrial dysfunction is associated with ALS [20].

Model systems like yeast, *C. elegans*, drosophila, zebrafish, and mice could capture many aspects of ALS pathogenesis. Omic studies like transcriptomic, proteomic, and metabolomic studies on the model systems show deregulated pathways associated with the disease [7]. Studies using these models have enabled the analysis of ALS pathogenesis and its association with deregulated pathways [21]. Gene knock-down and knock-out studies are imperative to validate the role of deregulated pathways in protein aggregation and disease [22]. Genome-wide analysis using siRNA, or yeast knock-out or over-expression libraries have proved to be critical to understanding the role of genes with either protein aggregation or its toxicity [23]. Using a yeast model, a role for deregulated pathways in the aggregation of mutant Huntington’s has been demonstrated [24]. Yeast is used as a good model system to understand protein aggregation in many amyloid diseases, including ALS [25]. Studies using yeast have shown critical pathways that are deregulated on the expression of SOD1, FUS, or TDP-43 [25]. Previous studies using the yeast model of FUS and TDP-43-mediated ALS have shown the role of genes in deregulated pathways as modifiers of protein aggregation or toxicity [3]. Previous studies have demonstrated a role for oxidative phosphorylation in FUS, and TDP-43 aggregation in the yeast model of ALS [26]. Further, network-based pharmacology approach has been used to evaluate the role of flavonoids and ginsenosides on FUS and TDP-43 aggregation in ALS [27]. Taken together, the yeast model could be used to understand the role of deregulated pathways on protein aggregation and evaluate them as potential therapeutic targets in ALS.

In this study, we carried out an integrated transcriptomic and metabolomic analysis of the yeast model of TDP-43-induced ALS to elucidate pathways that are common to both transcriptomic and metabolomic results. This data was then compared with the transcriptomic data analyzed from the mice model of TDP-43-induced ALS from the GEO database. Further, we compared the metabolic pathways obtained in yeast with those obtained for transcriptomic ALS patients brain datasets. The metabolomic results were also compared in turn with mice and human datasets. The comparative analysis helped to elucidate highly conserved pathways common to both model systems. Further, metabolic addition experiments and gene knock-out studies were used to validate the role of deregulated pathways in amyloidogenesis. We have performed similar studies on other metabolic and auto-immune diseases such as Huntington’s disease, glaucoma, rheumatoid arthritis, and avascular necrosis [24,28,29,30,31]. Our current study showed that fatty acid degradation, TCA cycle, nicotinate, and nicotinamide metabolism as the major perturbed pathways that could modulate amyloidogenesis in TDP-43-induced ALS. The results are discussed in light of their relevance to TDP-43 aggregation and disease. The integrated-omic analysis of the yeast model concomitant with its comparison with the patient or mice model, and the validation by yeast knock-out strain holds great promise for understanding the biology of ALS and elucidating potential therapeutic targets in the disease.

## 2. Materials and Methods

**Transformation and induction of TDP-43 plasmids in Saccharomyces cerevisiae:** The TDP-43 plasmids (TDP-43-WT, TDP-43-Q331K, TDP-43-M337V, TDP-43-G294A) used for the study were acquired from Adgene. The details of different plasmids used for the study are provided below (Table 1).

BY4741 (MATα his3Δ1 leu2Δ0 met15Δ0 ura3Δ0) and BY4742 (MATα his3Δ1 leu2Δ0 lys2Δ0 ura3Δ0) were used as model systems. Transformation was carried out using standard electroporation protocols using Bio-rad Gene Pulser Xcell and default yeast settings (1.5 KV/25 uF/200 Ω). Ura (Himedia) selection was used as a selective medium. YNB (Himedia Yeast Nitrogen Base) with aminoacids and Himedia glucose were used as energy supplementations. Plates were incubated at 30 °C. Transformed colonies grew after 2–3 days of transformation. The genes of interest were under galactose promoter. Transformed colonies were grown in Himedia Ura-YNB dextrose media for 12 h. The cells were then washed with PBS (phosphate buffer saline-pH 7) and grown in raffinose medium (Himedia Ura-YNB-raffinose) for 6 h. Cells were again washed with PBS and treated with galactose medium (Himedia Ura-YNB-galactose). After 8 h of induction, cells were harvested for omic analysis.

**Fluorescence imaging and sample preparation**: Induced Cells were observed under a Laben fluorescence microscope at 100× magnification. TDP-43 protein was tagged with E-YFP, which has an excitation wavelength of 510 nm and emission at 535 nm. The yeast cells were then illuminated with an appropriate fluorescence LASER, and the images were captured. Imaging was carried out in dark field and bright field settings. Images were arranged using PhotoScape 4.2.1 [32] (http://www.photoscape.org/ps/main/index.php and Microsoft PowerPoint software. Flow Cytometric Analysis and fluorescence quantification were carried out using ImageJ software (Version 1.46) [33]. Flow Cytometry was carried out using the Beckman Coulter Flow Cytometer machine. Cells were washed with phosphate buffer saline (pH 7.4) and injected into Flow Cytometer. A plot between forward scatter and side scatter was made to identify healthy living cells. The healthy cells were gated and used for the remaining analysis. The identified singlet cells were further analyzed using FL1 and FL2 LASERS to identify pure E-YFP expressing cells. Yeast cells were then counted using haemocytometer. Around 45 million cells were used for RNA sequencing, and 8 million cells were used for metabolomics.

**Metabolomics:** Metabolomics was carried out to understand the metabolic profile of Saccharomyces cerevisiae transformed with TDP-43 and its mutant. Eight million yeast cells were aliquoted in quadruplicates for each sample. Sample preparation was carried out using standard protocols, and the cell pellets were spiked with internal standards. Analysis was carried out using Waters X-Bridge amide 3.5 µm, 4.6 × 100 mm column (positive and negative ionization mode). Positive ionization mode was carried out for TDP-43-Q331K, TDP-43-M337V and TDP-43-G294A, while negative ionization mode was carried out for TDP-43-Q331K. The Agilent 6490 iFunnel triple quadrupole LC/MS system was used for further analysis. Peak intensities of pooled samples and human serum were used for quality control. The machine parameters, solvent details, MRM transitions for detected metabolites, and peak intensity table for different comparisons are provided in the supplementary section (Table S4). Post-processing was carried out using Agilent Mass Hunter and Metabo-analyst (www.metaboanalyst.ca, Version 5.0, January 2022–March 2022) [34]. Principal Component Analysis was performed to identify the similarity between the samples. Significant metabolites were identified using FDR corrected *p*-value of 0.25 and Log2 Fold change. Pathway analysis was carried out using the same website (www.metaboanalyst.ca, Version 5.0, January 2022–March 2022).

Transcriptomics (Library preparation and RNA sequencing): RNA Sequencing of Saccharomyces cerevisiae transformed with TDP-43 plasmids: RNA isolation involves the breaking of yeast cell wall and the isolation of cellular RNA. A freeze-thaw approach was used for lysing the cell wall. Three freeze-thaw cycles were carried by dropping the yeast pellets in liquid nitrogen for a minute and keeping it in ice the next minute (1 cycle). After 3 cycles, the cell wall was lysed further by vigorous vortexing (not too vigorous). Further, Total RNA was extracted using the standard Trizol method. The integrity of the isolated RNA was checked using Agilent Tape Station 2100 and quantification was carried out using the Qubit method. mRNA libraries were prepared using the NEBNext^®^ mRNA Library Prep Reagent Set for Illumina. Briefly, mRNA was isolated using oligo-DT beads (NEB Next Poly(A) mRNA Magnetic Isolation Module (E7490)) followed by heat fragmentation, cDNA conversion, and adaptor ligation. Adaptor ligated libraries were size selected using ampure beads. Sequencing was done using Illumina Hiseq2500 to obtain 100 bp paired end reads. Each sample had reads >10 million. Once the fastq files were obtained, they were subjected to a quality check using Fastqc tool [35]. The reads were then aligned to the reference *Saccharomyces cerevisiae* S288C strain (Downloaded from The University of California, Santa Cruz (UCSC) genome browser) using bowtie2 [36] with default parameters. A binary alignment map (BAM) file was obtained using Samtools [37]. Saccharomyces cerevisiae S288C strain-specific annotation file was downloaded from UCSC and read counts were generated using bedtools. The DESeq2 package from R [38] was used to obtain differentially expressed genes (Table S8). Significant differentially expressed genes [Adj.*p*-Value < 0.05 and fold change cut-off of +1 and −1 (Log2)] were used for pathway enrichment analysis. Pathway enrichment analysis was carried out using Enrichr [Analysis date: January 2022–March 2022 (www.maayanlab.cloud/Enrichr)] [39,40,41], and Network Analyst (www.networkanalyst.ca, Version 4.0) [42] was used for GSEA studies. Enrichr identifies key paths using a combined score. The combined score is calculated by multiplying the log of the *p*-value obtained from the Fisher exact test by the Z-score of the departure from the predicted rank. The top pathways with a high combined score are significant (Generally more than 10). The raw data has been deposited in the NCBI GEO database (www.ncbi.nlm.nih.gov/bioproject/817798).

**RT Polymerase Chain Reaction (RT-PCR):** RT-PCR was performed to validate the results obtained from transcriptomics. Three genes that were expressed in the TCA cycle, signaling, and fatty acid metabolism (CIT3, MIH1, FAA2) were identified. cDNA synthesis was performed using the Thermo Scientific cDNA synthesis kit (#AB-1453/B) as per the manufacturer instructions. PCR was performed using 384 well format Quant Studio 5 PCR machine. Primers were designed using Primer Blast, and synthesized by Barcode Biosciences (Juniper Life sciences Pvt Ltd., Bengaluru, India). ALG9 was used as a housekeeping gene. The primer sequences used for the study are presented below (Table 2). Further, the result of melt curves is provided in the supplementary section (Table S7). The experiment was performed in triplicates with 3 biological replicates, and the significance was computed.

**Integrated pathway analysis (Joint Pathway Analysis):** RNA sequencing results and Metabolomic results were integrated using Metabo-analyst (www.metaboanalyst.ca, Version 5.0) [43]. A list of significant genes and metabolites was uploaded in Metabo-analyst 5.0. Enrichment analysis involves the combined enrichment of significant genes and metabolites. The hyper-geometric method of enrichment analysis was used. Integration was performed using the option “All Integrated Pathways”. The significant pathways were identified with an FDR correction of 0.01.

**GEO (Gene Expression Omnibus) data analysis:** Gene Expression dataset pertaining to mice motor neuron expressing TDP-43 aggregates and its mutant were identified from Gene Expression Omnibus Database (**GSE111775**) [14]. Appropriate controls and samples (2 A315T Mutants + 2 TDP-43 Wild Type Controls) were selected. Network Analyst and KEGG were used for GSEA Analysis date: January 2022–March 2022 analysis [42,44,45,46]. A GEO dataset pertaining to human post-mortem ALS cortex sections (**GSE124439**) [47] was also used for the study (146 disease samples + 16 controls). Differential gene expression and Gene Set Enrichment Analysis was carried out using Network Analyst [42].

**Comparative analysis:** Commonality analysis was carried out between the GEO-derived dataset of TDP-43-A315T, ALS Post-mortem cortex sections, and the integrated analysis pathway results of the TDP-43-Q331K Mutant. A Venn diagram was plotted using Venny (www.bioinfogp.cnb.csic.es/tools/venny, version 2.1, Analysis date: January 2022–March 2022). Commonality analysis was also carried out between literature-derived metabolomic datasets of human Cerebrospinal fluid (CSF) [48], Mice TDP-43-A315T motor neurons [49], and pooled pathways obtained from yeast TDP-43 mutants. The Venn diagram was made using (www.bioinfogp.cnb.csic.es/tools/venny, Version 2.1, Analysis date: January 2022–March 2022).

**Experimental validation (Metabolite addition experiments and knock-out studies):** Metabolite addition experiments have been carried out on various model systems [50]. We carried out experiments on *Saccharomyces cerevisiae* transformed with TDP-43 and its mutants. Transformed cells were grown in URA-YNB-Dextrose medium for 12 h. The cells were then pelleted, washed, and grown in URA-YNB-Raffinose medium. After 7 h, the cells were again pelleted, washed, and grown in galactose medium. The cells were treated with different metabolites based on the transcriptomic and metabolomic results. The cells were allowed to grow in galactose medium for 8 h. After 8 h, fluorescence imaging was carried out as stated before. Samples were randomized for imaging studies. Further, fluorescence quantification was carried out using Icy software [51] (icy.bioimageanalysis.org, version 2.1.0.0). The results were analyzed using Microsoft Excel 2019. The yeast knock-outs library from Dharmacon (Catalog no: YSC1021) was used for the study’s yeast knock-outs. Yeast knock-outs were transformed and imaged in a similar manner as described previously. Fluorescence quantification was performed using Icy software (icy.bioimageanalysis.org, version 2.1.0.0) and Microsoft Excel 2019.

**Protein preparation and Filter Retardation assay:** Galactose-induced cells were centrifuged for 5 min at 6000 rpm and then rinsed in sterile double distilled water. Total protein, soluble, and insoluble fraction were purified using standard protocols [52]. The yeast pellets were subjected to three freeze-thaw cycles using liquid nitrogen, with each phase lasting for 5 to 10 min. After 3 complete cycles, 200 µL of lysis buffer (30 mM Merk Tris-HCl, 200 mM NaCl, 2 mM Merk EDTA, 5% glycerol- pH-8) and acid washed glass beads (G8772-10G) were added to the pellets, and 10 cycles of homogenization was carried using Bertin Precellys Evolution Super Homogenizer. The lysates were transferred to a new tube and a quick spin was carried out to remove the cell wall components. Total protein was obtained by centrifugation for 20 min at 13,000 rpm, with the clear cell lysate being transferred to a new 1.5 mL microfuge and kept at 4 °C. Soluble and insoluble fractions were separated using an Ultra-centrifugation process. The total protein was spun at 17,000× *g* for 20 min at 4 °C. The supernatant with soluble fraction was removed and transferred to another microfuge. The soluble protein was stored in −80 °C. The pellet was redissolved in 2% SDS. Mild sonication was carried out to completely dissolve the pellet. The mixture was again centrifuged 17,000× *g* for 20 min and the supernatant containing insoluble fraction was stored in −80 °C. The protein concentrations of the fractions were estimated. Filter retardation was carried out using established protocols [53]. The blot was blocked for 2 h with 25 mL of 5% casein in TBST. On a gel rocker, the blot was washed twice with 25 mL of TBST (Tris Buffer saline with Tween-20). The pre-soaked nitrocellulose membrane in 2 percent (*w*/*v*) SDS containing TBS buffer was used to assemble the bio-dot device (0.2 m, Cat. No: 10600016, GE Health Care). 150 µg of protein was heated at 100 °C for 10 min and was loaded in the wells. A vacuum was applied to enhance the binding of the protein with the membrane. After 10 min, the membrane was removed. The blot was incubated in 20 mL of TBST overnight at 4 °C with 2 µL of mouse anti-GFP antibody (B-2, sc-9996, Santa Cruz Biotechnology, Dallas, TX, USA). On a gel rocker, the blot was washed twice with 25 mL of TBST. The cleaned blot was incubated for 2 h at room temperature with 1 µL of goat anti-mouse IgG-HRP antibody (Invitrogen, SNN404Y) in 25 mL of TBST. The ECL substrate (K12045-D20, Advansta, San Jose, CA, USA) and Syngene Gbox F3 were used to create the blot. The blots were quantified using Icy software (icy.bioimageanalysis.org, version 2.1.0.0) and Microsoft Excel 2019.

## 3. Results

### 3.1. Metabolomic Analysis of TDP-43 Q331K Mutant Shows Deregulation of Pathways in the Yeast Model of TDP-43 Aggregation

**Figure 1:**
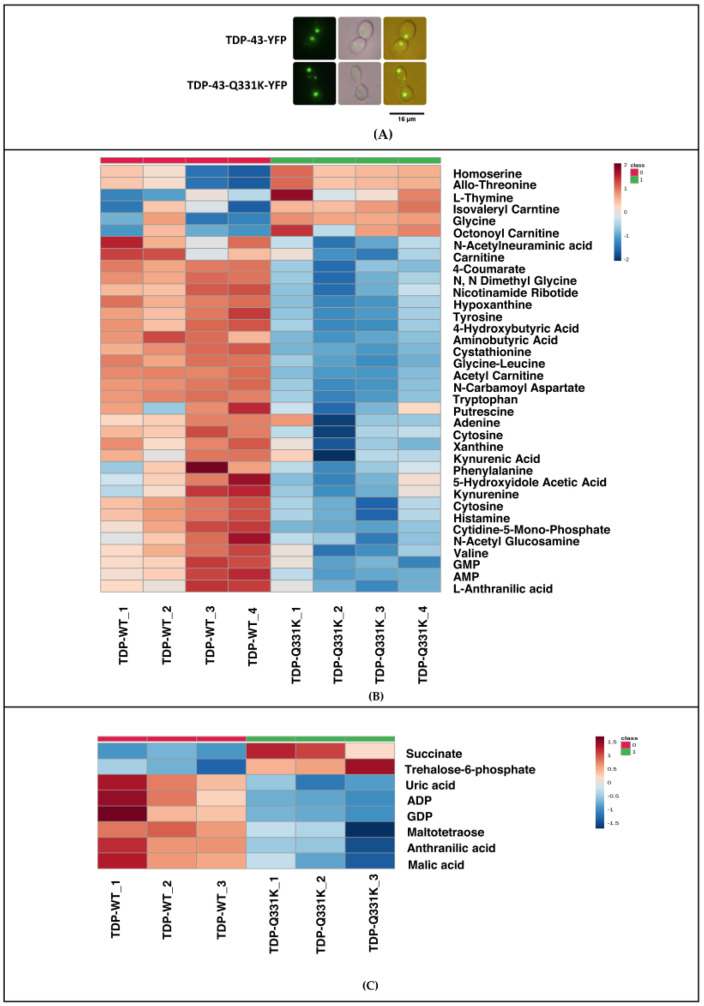
(**A**) Fluorescence images (dark field, bright field and overlay) showing *Saccharomyces cerevisiae* transformed with TDP-43 and its mutant. (**B**) Heat map representing differentially expressed significant metabolites obtained from TDP-43-Q331K Mutant (Water’s Amide Column-Positive Mode-4 biological replicates). (**C**) Heat map representing differentially expressed significant metabolites obtained from TDP-43-Q331K Mutant (Water’s Amide Column-Negative Mode-3 biological replicates) (Two Tailed *t*-test FDR corrected *p*-value of 0.25) (Figures were made using www.metaboanalyst.ca, Version 5.0).

TDP-43 wild type and Q331K Mutant tagged to EYFP were expressed in *S. cerevisiae* after transformation with respective plasmids (Figure 1A). Quality control for transformation was assessed using Flow Cytometry and fluorescence quantification studies. Consistent with previous reports, the fluorescent amyloid foci were significantly higher in the majority of mutants compared to the wild type (Figure S1).

A targeted metabolomic analysis of *S. cerevisiae* expressing TDP-43 wild type and Q331K Mutant was carried out. The metabolic profile of the TDP-43 mutant Q331K was compared to TDP-43 wild type. The experiments were carried out in 4 biological replicates, as described in the methods section. One hundred eighty metabolites were targeted, of which 77 were identified. Those metabolites with a CV (Coefficient of variation) of ≤20% after normalization with internal standards were used for further analysis. A total of 36 significant differential metabolites were obtained (Figure 1B). Using metabolite enrichment analysis in Metaboanalyst, the metabolites were further grouped into pathways, as described in the methods section. Principal Component Analysis-based clustering categorized the control and experimental datasets into two separate groups (Figure S3). Pathway analysis using Metaboanalyst grouped the metabolites into 28 pathways. A total of 108 metabolites were targeted in the negative mode for the TDP-43 Q331K Mutant, of which 47 metabolites were identified. Differential metabolites with a CV (Coefficient of variation) of ≤20% after normalization was taken for further analysis using Metaboanalyst. Eight significant differential metabolites were identified in a non-parametric analysis using an FDR (False Discovery Rate) correction of 0.25 (Figure 1C). Commonality analysis of pathways obtained for Q331K in positive and negative mode showed considerable overlap. Taken together, our results show significant metabolic deregulation in the TDP-43 Q331K Mutant.

### 3.2. Transcriptomic Analysis of the Yeast Model of TDP-43 Aggregation Expressing TDP-43 Q331K Shows Deregulated Metabolic and Signaling Pathways with Implications for Disease

**Figure 2:**
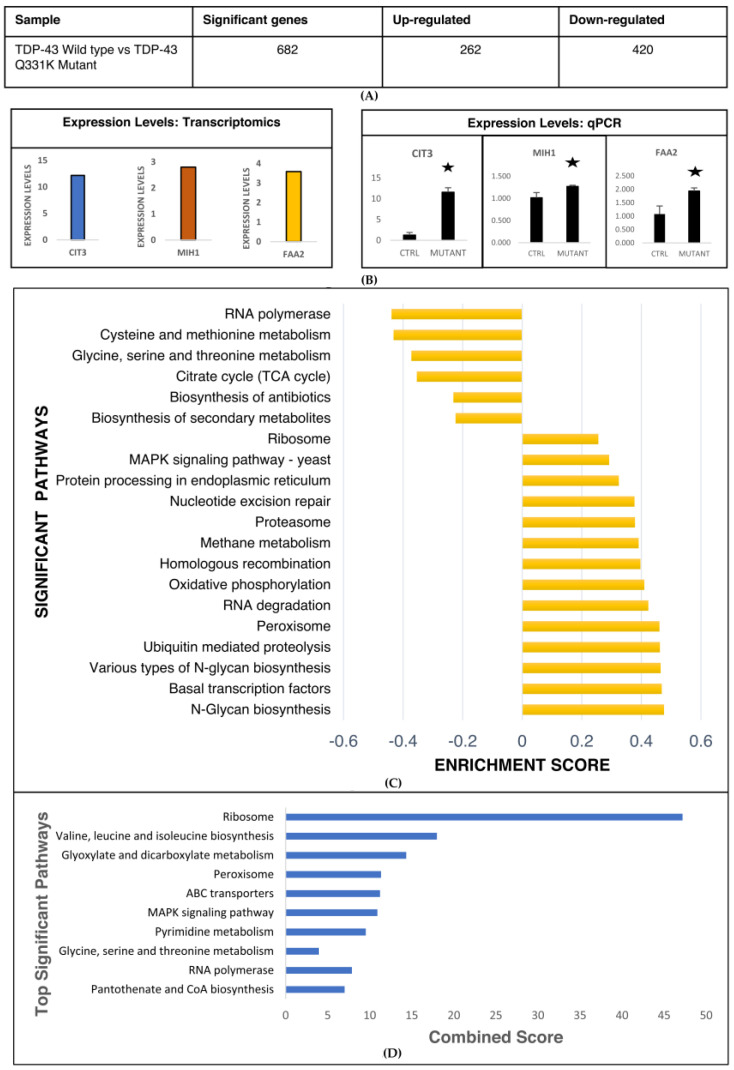
(**A**) Table representing details about number of significant genes obtained after transcriptomic analysis of TDP-43-Q331K Mutant (Adj.P-Value ≤ 0.05–3 replicates). (**B**) Bar graphs representing expression levels of CIT3, MIH1, and FAA2 from transcriptomics and RT-PCR. (**C**) Results representing GSEA results from RNA sequencing of *Saccharomyces cerevisiae* transfected with TDP-43-Q331K Mutant. (Figures were made using www.networkanalyst.ca (Version 5.0) and Microsoft Excel 2019). (**D**) Results obtained from pathway enrichment analysis of transcriptomic data obtained from RNA sequencing of *Saccharomyces cerevisiae* transfected with TDP-43 and its mutant using Enrichr and KEGG database (www.maayanlab.cloud/Enrichr and Microsoft Excel 2019).

**Figure 3:**
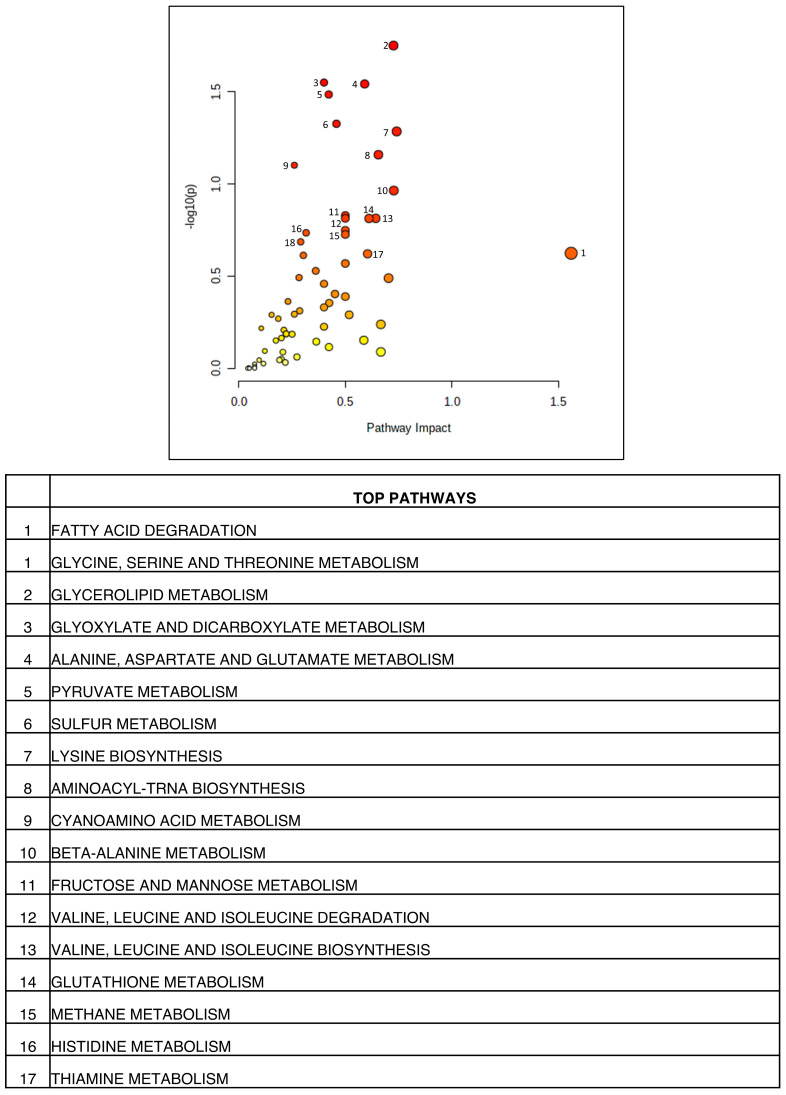
Results of integrated pathway enrichment analysis carried out for the enriched genes and metabolites. The colours in the graph represented gives the significance of the enriched pathways. Red represents high significance values and yellow represents lower significance levels. The size represents the impact of the pathway in the disease condition (Figures were made using www.metaboanalyst.ca, (Version 5.0)).

A transcriptomic analysis of the TDP-43 Q331K yeast model of TDP-43 aggregation was performed using 3 replicates, as described in the methods section. The analysis yielded 682 significant differentially expressed genes with fold change cut-off of +1 and −1 (Log2), and an adjusted *p*-value of ≤0.05 (Figure 2A). Of the total significant differentially expressed genes, 262 genes were upregulated while 420 genes were downregulated in the TDP-43-Q331K Mutant. (Figure 2A). To further validate the expression of genes observed in the transcriptomic analysis, we performed a RT-PCR of selected genes, as outlined in the methods section. The expression levels of CIT3, MIH1, and FAA2 in the mutant TDP-43 (Q331K) compared to wild type controls in the transcriptomic dataset was 12.6, 2.8, and 3.59-fold, respectively (Figure 2B). Our RT-PCR results for the genes CIT3, MIH1, and FAA2 corroborate well with the results obtained from the transcriptomic datasets (Figure 2B). Having performed the transcriptomic analysis using Enrichr, we validated the results using RT-PCR. Further, we performed a Gene Set Enrichment Analysis (GSEA) of the transcriptomic datasets of yeast expressing wild type and mutant TDP-43 (Table S8). Our analysis shows the deregulation of metabolic and signaling pathways (Figure 3C). The deregulated metabolic pathways include cysteine and methionine metabolism, TCA cycle, biosynthesis of secondary metabolites, methane metabolism, oxidative phosphorylation, peroxisome, and N-glycan biosynthesis, while signaling pathways include MAPK signalling, basal transcription factors, nucleotide excision repair, and ubiquitin-mediated proteolysis (Figure 3C). The significant differential genes were also binned into pathways using Enrichr (Figure 3D). The significant pathways that emerged from the Enrichr analysis include ribosome pathways, fatty acid degradation, metabolism of various amino acids (Cysteine and methionine, glycine serine and threonine, valine, leucine and isoleucine, lysine), peroxisome, pyruvate metabolism, and MAPK signaling (Figure 3D). Taken together, our transcriptomic pathway analysis using two different softwares shows considerable concordance of deregulated pathways. We have used two different softwares for the analysis, and despite the differences in methods employed, both softwares identified similar pathways. The common pathways were identified using a Venn diagram which is provided in Table S10. The results suggest that the deregulated pathways might have potential implications for the disease, which we have tried to validate using the yeast model system.

### 3.3. Integrated Analysis of Transcriptomic and Metabolomic Datasets from the Yeast Model of TDP-43 Aggregation Show Significant Pathways with Potential Implications for Disease

Integrated analysis of transcriptomic and metabolomic datasets from the yeast model of TDP-43 aggregation was performed using Metaboanalyst, as described in the methods section. The analysis yielded 18 significantly deregulated metabolic pathways (Figure 3). Our analysis shows deregulation of various pathways like fatty acid degradation, glycine, serine and threonine metabolism, alanine, aspartate and glutamate metabolism, lysine biosynthesis, cyano-amino acid metabolism, beta-alanine metabolism, valine, leucine and isoleucine degradation and biosynthesis, as well as histidine metabolism (Figure 3). The metabolism of glycerolipid and fatty acid degradation was deregulated. Further, glyoxylate and dicarboxylate, pyruvate, glutathione, sulfur, methane, thiamine, as well as fructose and mannose metabolism were found to be deregulated (Figure 3). Results of the integrative analysis show considerable concordance in pathways between transcriptomic and metabolomic datasets. These results suggest a potential role for metabolic pathways in ALS.

### 3.4. Metabolomic Analysis of Different TDP-43 Mutants Shows Deregulation of Similar Pathways in the Yeast Model of TDP-43 Aggregation

**Figure 4:**
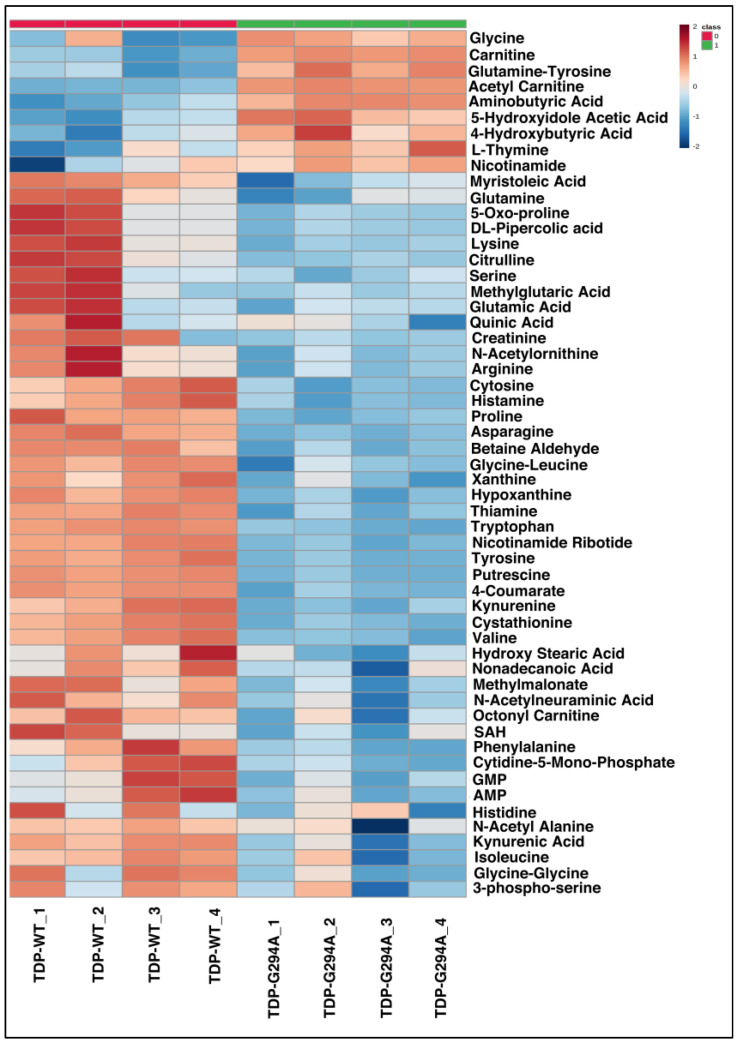
Heat map representing differentially expressed significant metabolites obtained from TDP-43-G294A Mutant (Water’s Amide Column-Positive Mode-4 biological replicates) (Figures were made using www.metaboanalyst.ca, Version 5.0).

**Figure 5:**
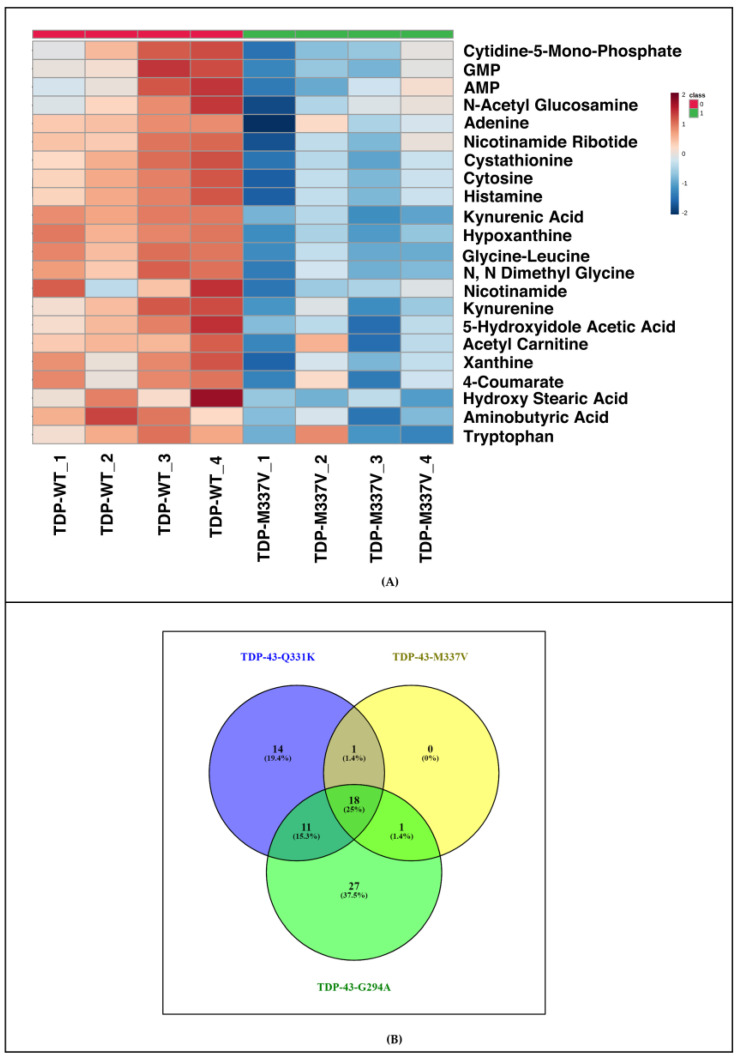
(**A**) Heat map representing differentially expressed significant metabolites obtained from TDP-43-M337V Mutant (Water’s Amide Column-Positive Mode-4 biological replicates) (Figures were made using www.metaboanalyst.ca, Version 5.0). (**B**) Common metabolites obtained between different TDP-43 mutants (Figure was made using: www.bioinfogp.cnb.csic.es/tools/venny version 2.1).

To understand if yeast expressing different TDP-43 mutants (G294A, M337V) exhibited deregulation of metabolic pathways which are similar to those expressing Q331K, we carried out targeted metabolomic analysis of *S. cerevisiae* expressing wild type or TDP-43 mutants (G294A, M337V). The metabolic profile of TDP-43 mutants (G294A and M337V) was compared to TDP-43 wild type. For G294A and M337V, a total of 57 and 21 significant differential metabolites were obtained (Figure 4 and Figure 5A). Pathway analysis using Metaboanalyst grouped the metabolites into 30 pathways in G294A and 8 pathways in M337V (Tables S1–S3). The metabolites obtained from the positive and negative mode analysis of Q331K were pooled. A total of 18 metabolites were common among all the TDP-43 mutant datasets, while 11 were common between Q331K and G294A, and only 1 metabolite was common between Q331K and M337V as well as G294A and M337V, respectively. A total of 14, 26, and 8 metabolites were unique to Q331K, G294A, and M337V datasets (Figure 5B). Since many metabolites are binned into similar pathways, we looked for metabolic pathways that are common among the various TDP-43 mutants.

**Figure 6:**
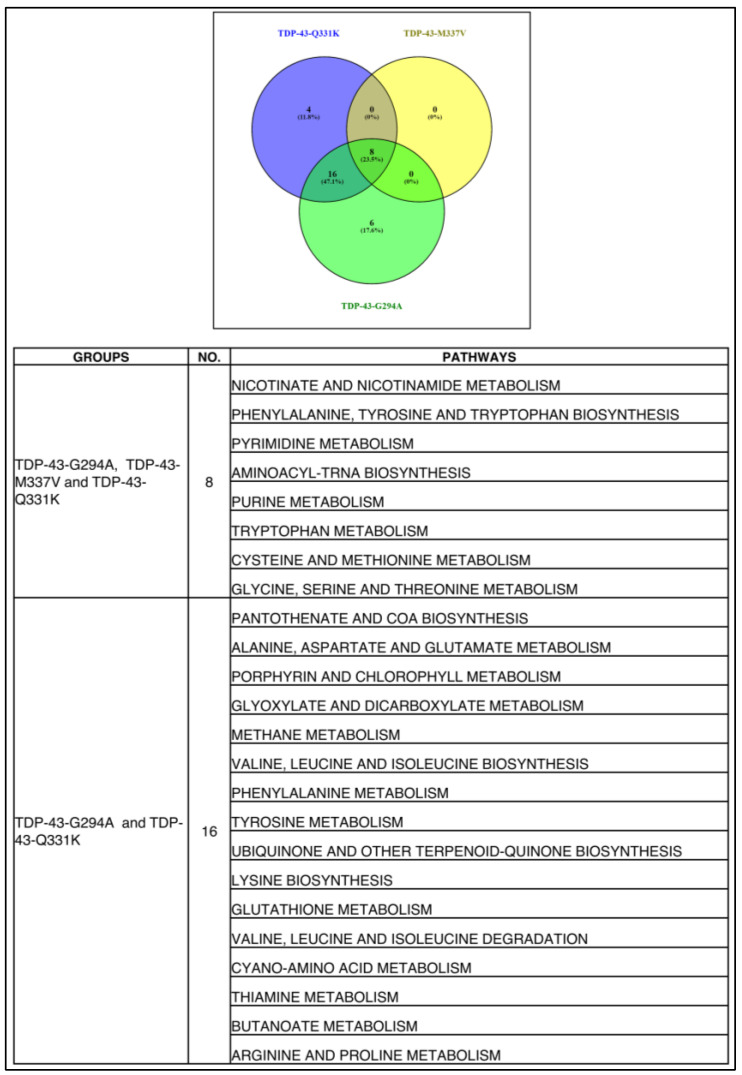
Venn diagram representing common metabolic pathways (metabolomics) enriched by significant metabolites between TDP-43-Q331K, TDP-43-M337V, TDP-43-G294A Mutant and KEGG database (Figure was made using: www.bioinfogp.cnb.csic.es/tools/venny, Version 2.1).

Commonality analysis for overlapping pathways yielded 8 pathways as common among all the TDP-43 mutants, while 16 pathways were common between Q331K and G294A (Figure 6). The TDP-43 mutants Q331K had 4 pathways, and G294A had 6 pathways unique to them (Figure 6). The list of common pathways is provided in Figure 6. A considerable overlap of deregulated metabolic pathways between different TDP-43 mutants was observed.

### 3.5. Analysis of Gene Expression Datasets from the Motor Neuron of the Mice Model of TDP-43 (A315T) and Post-Mortem Cortex of ALS Patients Shows Deregulation of Pathways with Potential Implications for Disease

We further asked if our findings in the yeast model are of relevance to ALS. For this, we carried out an analysis of transcriptomic datasets of mice models of ALS and ALS patients from the GEO database. The datasets used are the TDP-43 A315T expressing transgenic mice motor neurons and cortex of ALS patients. GSEA analysis of motor neuron dataset from mice model of ALS showed deregulation of pathways like primary immunodeficiency, neuroactive ligand-receptor interaction, Parkinson’s disease, glycerolipid metabolism, spliceosome, oxidative phosphorylation, and ribosome. The detailed results are provided in the supplementary section (Figure S6). Similarly, GSEA analysis of ALS patient datasets show deregulation of protein export, TCA cycle, Parkinson’s, Huntington’s and non-alcoholic fatty liver disease, longevity regulating pathway, Autophagy, and ubiquitin-mediated proteolysis (Figure S6).

### 3.6. Commonality Analysis of Pathways from Yeast TDP-43 (Q331K), Mice TDP-43 (A315T) and Human ALS Shows Deregulated Pathways Conserved across Taxa, Study Setting, and Platforms

**Figure 7:**
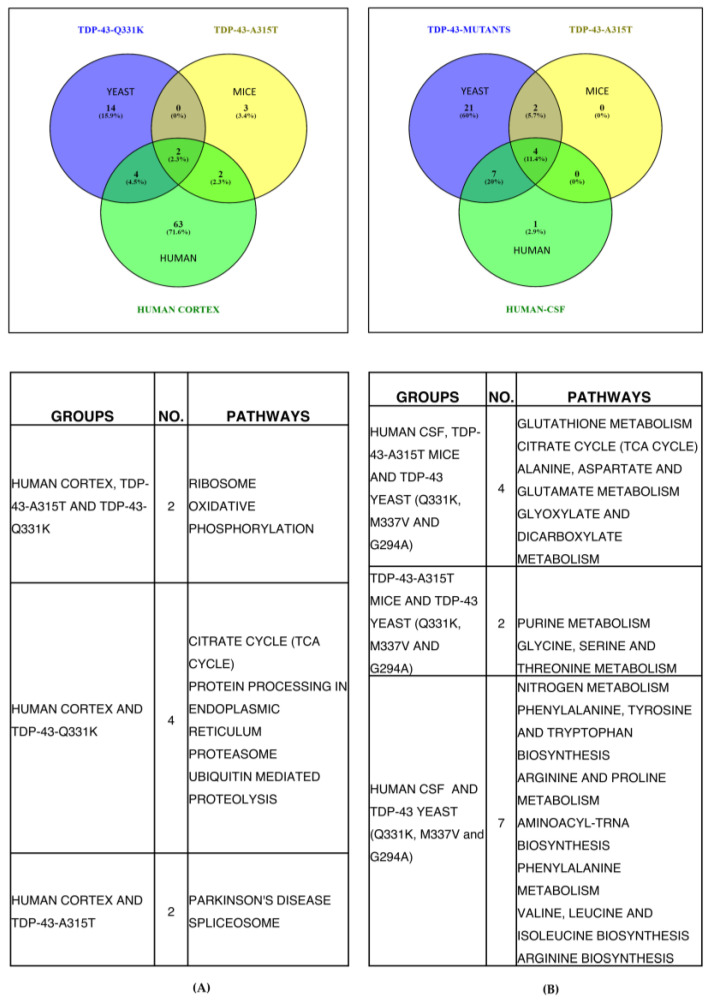
(**A**) Venn diagram representing common metabolic pathways (transcriptomics) enriched between TDP-43 A315T Mutant—Mice [GSE111775] and TDP-43 Q331K Mutant and ALS patient cortex sections [GSE124439] (KEGG database) (Figure was made using www.bioinfogp.cnb.csic.es/tools/venny, Version 2.1). (**B**) Venn diagram representing common metabolic pathways (metabolomics) enriched between TDP-43 A315T Mutant (Mice) and TDP-43 mutants (Q331K, M337V and G294A) and ALS patient CSF. (KEGG database) (Figure was made using www.bioinfogp.cnb.csic.es/tools/venny, Version 2.1).

Commonality analysis of deregulated pathways across ALS patients, yeast and mice models of ALS was performed. Two pathways (ribosome and oxidative phosphorylation) were common to yeast, mice, and human datasets (Figure 7A). A total of 4 pathways (TCA cycle, protein processing in ER, proteasome, and ubiquitin-mediated proteolysis) were common between yeast and human, while 2 pathways (Parkinson’s disease and spliceosome) were common between human and mice datasets (Figure 7A). The total pathways unique to ALS patients, mice, and yeast models of ALS were 63, 3, and 14 pathways, respectively (Figure 7A). Taken together, the results of overlapping pathways suggest their potential involvement in ALS.

Further, the metabolites obtained from literature for TDP-43-A315T transgenic mice motor neurons were analysed using Metaboanalyst, and the results are provided (Table S11). Metabolomic datasets of ALS patient CSF were also analysed using Metaboanalyst, and the results of deregulated pathways are provided (Table S12). Commonality analysis was also performed for pooled yeast metabolomic dataset from our study with TDP-43-A315T transgenic mice motor neurons and human CSF metabolomic datasets (Figure 7B). Our analysis shows 4 pathways were common among all three datasets. These pathways include glutathione metabolism, TCA cycle, alanine, aspartate, glutamate, glyoxylate, and dicarboxylate metabolism. The yeast and mice dataset showed two pathways to be common. These pathways include purine, glycine, serine, and threonine metabolism. The yeast and human datasets showed seven pathways to be common between them. These pathways include metabolism of nitrogen, phenylalanine, arginine and proline, as well as biosynthesis of phenylalanine, tyrosine, tryptophan, amino-acyl-tRNA, valine, leucine, isoleucine, and arginine. Only 1 pathway was unique to humans, while 17 pathways were unique to yeast. The unique pathways in yeast might be due to the targeted analysis of more metabolites compared to other systems. Overall, our results show considerable concordance among the datasets compared.

### 3.7. Metabolic Addition Experiments and Gene Knock-Out Experiments in the TDP-43 Yeast Model of TDP-43 Aggregation Reiterate a Role for Deregulated Pathways in the Disease Process

**Figure 8:**
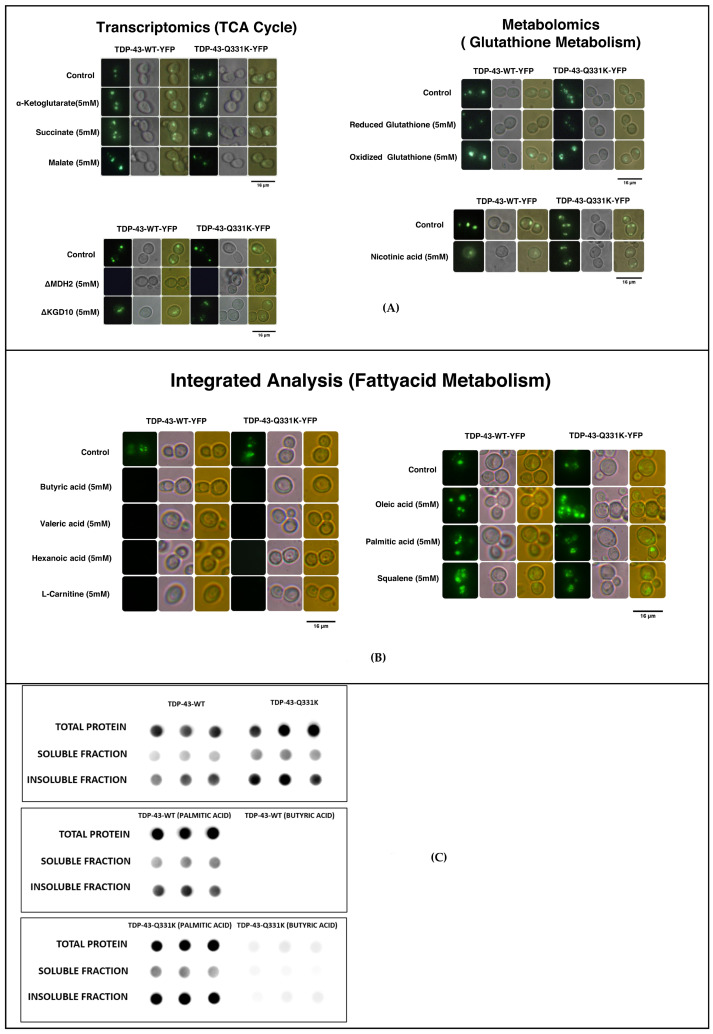
(**A**) Results of metabolite addition experiments show that TCA cycle metabolites increase amyloidogenesis in *Saccharomyces cerevisiae* transformed with TDP-43 and its mutant. ΔKGD10 and ΔMDH2 reduced amyloidogenesis. Fluorescence quantification results are provided in Tables S14–S21. Results of metabolite addition experiments (dark field, bright field and overlay) show that reduced glutathione reduced amyloidogenesis, while oxidized glutathione increased amyloidogenesis in *Saccharomyces cerevisiae* transformed with TDP-43 and its mutant. Treatment with nicotinic acid was found to attenuate amyloidogenesis. Fluorescence quantification results are provided in Tables S14–S21. (**B**) Imaging results (dark field, bright field and overlay) of metabolite addition experiments show that short-chain fatty acids reduce amyloidogenesis, while long-chain fatty acids increase amyloidogenesis in *Saccharomyces cerevisiae* transformed with TDP-43 and its mutant. (**C**) Figure representing images of filter retardation assay carried out on treated *Saccharomyces cerevisiae* transformed with TDP-43 and its mutant. Butyric acid showed complete absence of aggregates, while palmitic acid showed increased protein aggregates.

Our integrative analysis of transcriptomics and metabolomics shows deregulation of the TCA cycle. Similarly, the TDP-43 mice model brain and ALS patient post-mortem cortex transcriptomic data also displayed deregulation of the TCA cycle. Metabolic addition experiments using succinate and alpha-ketoglutarate show significantly elevated protein aggregates (Figure 8A and Figure S18). Consistent with these observations, KO of KGD10 (alpha-ketoglutarate dehydrogenase) and MDH2 (a cytosolic isoform of malate dehydrogenase) significantly reduced amyloid formation (Figure 8A and Figure S18). Previous studies have shown that increased fumarate leads to the reverse reaction in mitochondrial succinate dehydrogenase complex II. The reverse reaction can lead to elevated levels of Reactive Oxygen Species (ROS). Furthermore, our metabolic data shows that the levels of malate were significantly lower in the TDP-43 mutant compared to the wild type. However, the addition of malate to the TDP-43 wild type or mutant yeast model did not result in any reduction in amyloidogenesis.

The transcriptomic data from our yeast studies and the mice brain data from the GEO database show deregulation of glutathione metabolism. Supplementation of reduced glutathione significantly reduced amyloidogenesis, while oxidized glutathione led to an insignificant increase in amyloidogenesis (Figure 8A and Table S20). Metabolomic analysis of the yeast model shows deregulation of nicotinate and nicotinamide metabolism. Nicotinate and nicotinamide are important intermediates in the biosynthesis of NAD. The addition of nicotinate leads to a significant decrease in amyloidogenesis (Figure 8A and Table S20). Our results show that deregulation of the TCA cycle could lead to the production of ROS. Homeostatic mechanisms that scavenge ROS can potentially help to curtail protein aggregates in ALS. Our commonality analysis data show the deregulation of fatty acid metabolism in ALS. The role of short-chain and long-chain fatty acids has different implications in many neurodegenerative diseases [54]. Hence, metabolic addition experiments were carried out using short-chain fatty acids such as butyric, valeric, and hexanoic acid (Figure 8B). The results of imaging and quantification studies show a significant reduction in amyloid formation in the presence of short-chain fatty acids. The addition of long-chain fatty acids like palmitic and oleic acid or squalene, an intermediate in cholesterol biosynthesis, significantly increased amyloidogenesis in the yeast model of TDP-43 aggregation (Figure 8B and Table S16). Critical metabolite, which is essential for fatty acid metabolism like carnitine, significantly attenuated amyloidogenesis (Figure 8B and Table S14). Taken together, our results show that short-chain fatty acids and carnitine significantly impaired amyloidogenesis. The long-chain fatty acids were detrimental and might be correlated with the poor prognosis of the disease condition.

Further, we carried out filter retardation assay to quantify the wild type or mutant TDP-43 in the total protein, as well as in the soluble and insoluble fractions, as described in the methods section. In particular, we had used the wild type and mutant TDP-43 which are untreated or treated with butyric acid or palmitic acid. This will help to correlate the changes in total protein, and soluble and insoluble protein of TDP-43, as well as validate that the observations of fluorescence imaging experiments are reliable. Consistent with fluorescence imaging data, our analysis showed significantly higher total protein in the mutants compared to the wild type. Similarly, the amount of mutant TDP-43 in both soluble and insoluble fractions were significantly higher compared to the wild type (Figure 8C). We further asked if addition of short-chain fatty acids like butyric or palmitic acids changes the levels of TDP-43 wild type and mutant in the soluble and insoluble fractions. Consistent with our fluorescence imaging data, the results of palmitic acid addition showed elevated levels of TDP-43 in the total protein, and soluble and insoluble fractions in both wild type and mutant sets (Figure 8C). Similarly, the addition of butyric acid attenuated TDP-43 in wild type, and significantly reduced TDP-43 in mutant sets in the total protein, and soluble and insoluble fractions. The results are quantified with respect to controls, and are provided in Table S22. Taken together, these results demonstrate considerable concordance between the results of fluorescence imaging data and filter retardation assay. The overall work flow and summary of the results of the study are provided in Table S24.

## 4. Discussion

In this study, we used the yeast model of TDP-43 aggregation to understand the transcriptomic and metabolomic changes as a consequence of mutation in TDP-43. The relevance of deregulated pathways obtained for yeast were compared with those obtained for motor neurons from the TDP-43 mice model of ALS (GSE111775) and ALS patients (GSE124439). Further, the role of deregulated transcriptomic and metabolomic pathways on the aggregation of TDP-43 in the yeast model was studied. Furthermore, metabolic addition experiments and yeast knock-out of specific genes in deregulated pathways were used to validate their role in the disease progression. Consistent with previous reports, in the TDP-43 expressing yeast model elevated TDP-43 aggregation was observed in the mutant (Q331K, M337V and G294A) compared to the wild type, which also corroborates with results of the filter retardation assay. Previous studies have also shown increased severity and aggregation in TDP-43 mutants compared to the wild type TDP-43 expressing cells [55].

Transcriptomic analysis of yeast expressing mutant TDP-43 shows enrichment of peroxisome, ribosomes, metabolism of different amino acids, nucleotide metabolism, fatty acid metabolism, and MAPK signalling. Transcriptomic analysis of zebrafish transgenic for TDP43 (G348C) showed changes in the levels of many differentially expressed genes that were related to neuromuscular disorders, including ALS and muscular dystrophy [55]. The genes were related to calcium signalling, and mitochondrial and oxidative stress [55]. Knock-down of TDP-43 in mice neuronal models showed differentially expressed genes belonging to the GO terms, such as a response to an organic substance, regulation of apoptosis, cell adhesion, MAPKKK cascade calmodulin-binding, etc. [56]. Our GSEA analysis in the mice model showed that the pathways enriched include primary immunodeficiency, neuroactive receptor-ligand interaction, Parkinson’s disease, glycerolipid metabolism, spliceosome, oxidative phosphorylation, and ribosome. Similarly, in humans, GSEA analysis showed enrichment of genes involved in the protein life cycle, including autophagy and the proteasome pathway. The other pathways include the TCA cycle, Huntington’s, Parkinson’s, and non-alcoholic fatty liver disease, and signalling pathways. The yeast, human, and mice datasets exhibited an overlap of 2 pathways, which included ribosome and oxidative phosphorylation. Previous studies in our lab have shown that oxidative phosphorylation modulated TDP-43 aggregation in the yeast model of ALS [26]. Similarly, flavonoids from Ginseng were also shown to target the MAPK pathway [27]. Overall, the deregulated pathways show enrichment of metabolic and signalling pathways, which might have implications for protein aggregation and disease.

The increased ROS in TDP-43 mutants has been reported previously [57]. Previous studies using yeast and other model systems of ALS have revealed a role in mitochondrial dysfunction and associated metabolic rewiring in the disease [58]. Our previous analysis showed oxidative phosphorylation as a common deregulated pathway. Further, inhibitors of complex III and IV, or the knock-out of genes in these complexes (QCR8 and COX8), significantly reduced protein aggregation [26]. The mitochondrial dysfunction results in the generation of ROS, which is also associated with oxidative stress [59]. Consistent with this, oxidative stress is implicated in disease progression. The expression of mutant TDP-43 enhanced oxidative stress compared to the wild type expressing yeast cells [60]. The increased oxidative stress is also corroborated with elevated SOD1 in the previous study. Our metabolomic analysis of the yeast model showed changes in the metabolism of multiple amino acids, TCA cycle, starch and sucrose metabolism, pyruvate metabolism, as well as nicotinate and nicotinamide metabolism.

The integrative analysis of transcriptomic and metabolomics shows that the transcriptomic changes translate into metabolic changes in the yeast model of TDP-43. Previous studies have shown increased glycolysis and metabolites belonging to the TCA cycle in ALS [15]. To corroborate the role of deregulated metabolic pathways in amyloidogenesis, we used the yeast model to carry out metabolic addition experiments or gene knock-outs experiments. Previous studies using yeast KO and over-expression libraries have helped to conjure novel pathways that modulate amyloidogenesis [61]. Fatty acid supplementation has been shown to suppress the glycolytic pathway, and has been suggested to aid favourable prognosis in ALS [62]. The addition of short-chain fatty acids resulted in reduced protein aggregates in both wild type and mutant TDP-43 expressing yeast cells. The fluorescence and filter retardation assay shows a reduction of TDP-43 wild type and mutants in the short-chain fatty acid treated sets, and an increase in the long-chain fatty acid treated sets, compared to untreated controls. Short-chain fatty acids are shown to easily cross the blood-brain barrier, and are taken up by the neurons [63]. Studies using triglycerides, as well as the keto diet, have shown to have favourable consequences in ALS [64]. Consistent with this, supplementation of culture with carnitine—which helps in the transport of fatty acids mitigate amyloidogenesis. Carnitine supplementation has been shown to mitigate amyloids and improve performance in neurodegenerative diseases [65]. Glycolytic pathways also result in an increased NADH/NAD ratio [66]. NAD is a cofactor in many enzymatic reactions that are important for neuronal function and survival [67]. Our results show that the addition of nicotinic acid, an intermediate in the biosynthesis of NAD, mitigated amyloids in both wild type and mutant TDP-43 expressing yeast cells. Previous studies have also shown that supplementation of nicotinamide results in the clearance of amyloids in the mice model of amyloid disease [68]. Similarly, elevated NAD levels favoured neuronal survival [69]. Our results show a potential role for intermediates in NAD biosynthetic pathway in mitigating ALS progression.

Further, transcriptomic analysis of yeast and mice models of ALS shows deregulation of the glutathione pathway and oxidative stress, while transcriptomic and metabolomic analysis shows deregulation of the pentose phosphate pathway. Previous studies have also shown oxidative stress and ROS in the yeast model of TDP-43 aggregation [70]. Supplementation of reduced glutathione attenuated amyloid formation. Further, in vivo, the levels of NADPH are critical in maintaining the reduced glutathione pool [71]. Hence, reactions that are important for the generation of NADPH might have a favourable consequence to maintain redox homeostasis. Our metabolomics data shows reduced malate, an intermediate in the TCA cycle. However, supplementation of malate did not mitigate the amyloids in the yeast model system, but led to a slight increase in amyloid formation. Consistent with this observation, the knock-out of the cytosolic enzyme MDH2 mitigated amyloidogenesis. A previous study has shown similar results in the cell culture model of neurodegenerative disease [72]. The rescue was shown to be independent of increased ATP synthesis through glycolysis or oxidative phosphorylation [73]. The increased NADH resulting from mitochondrial dysfunction might reduce the availability of NAD for the biosynthesis of NADP [73]. Further, our transcriptomic and integrated analysis of transcriptomic and metabolomic data shows deregulation of single carbon metabolism. The pathway was found to be especially critical during the impaired pentose phosphate pathway, which is one of the major contributors to NADPH in cells [74]. As per previous studies, the NADPH biosynthesis pathway emerged as a top hit in a screen where glucose is not the major carbon source [75]. The absence of glucose might compromise the functioning of the pentose phosphate pathway, resulting in reduced NADPH [74]. The present work shows a potential role for the oxidative stress pathway in TDP43 aggregation, and the keto diet with short-chain fatty acids, in conjunction with antioxidants and nicotinate, might help to achieve a favourable prognosis in the disease. The above study was carried out using the yeast model system. However, with the robustness of the yeast model system, the data provided clearly indicates the role of short-chain fatty acids in clearing protein aggregates associated with ALS. Though yeast is a robust system, with 30 percent of the yeast genome being similar to humans, it also has many short comings [7]. The metabolic pathways in yeast are not completely homologus to humans. Further, the mitochondrial complex I is not well developed. Despite these differences, yeast has served as a good model system to understand gene/metabolite-amyloid interactions [24]. Hence, due caution should be exercised while interpreting results, and additional experiments with mammalian culture systems and mice models might be possible to validate the results.

## 5. Conclusions

The current study focuses on an integrative multi-omic analysis of the yeast model of TDP-43 aggregation for the identification of deregulated pathways, and comparing it with patients and other model systems. Further, the role of these deregulated pathways in amyloid formation in the yeast model of TDP-43 aggregation is discerned using metabolic addition experiments and gene knock-outs. Metabolomic analysis of TDP43 wild type or different mutants expressed in the yeast model of TDP-43 aggregation shows deregulation of metabolic pathways. These pathways include nicotinate and nicotinamide metabolism, glutathione metabolism, and metabolism of various amino acids. Transcriptomic analysis of the yeast model of TDP-43 aggregation (Q331K) shows deregulation of the TCA cycle, oxidative phosphorylation, peroxisome, fatty acid degradation, metabolism of amino acids. The integrated transcriptomic and metabolomic analysis shows glutathione pathway, fatty acid degradation, metabolism of various amino acids. The transcriptomic and metabolomic analyses displayed considerable concordance. Further, pathways from transcriptomic datasets of the yeast model were compared with those obtained from ALS patients and mice models of ALS from the GEO database. Our analysis shows that the TCA cycle, oxidative phosphorylation, as well as protein processing and degradation are common to different datasets. Validation of the results using the yeast model showed short-chain fatty acids significantly abrogated amyloid formation, while long-chain fatty acids significantly ameliorated it. Furthermore, results of the filter retardation assay of short-chain and long-chain fatty acids treated and untreated wild type and mutant cells corroborated well with the fluorescence image data. Similarly, reduced glutathione and succinate reduced protein aggregation. Consistent with this, KO of MDH2 and KGD10 reduced protein aggregation. Taken together, our results show deregulated pathways modulate amyloid formation in the yeast model of TDP-43 aggregation. The results show a potential use for a short-chain fatty acid-containing keto diet, and antioxidants such as glutathione and nicotinate in managing and improving the living conditions of ALS patients.

## Figures and Tables

**Table 1:** Table representing the details of different plasmids used for the study.

Plasmid ID	Strain Features
27447	pRS416Gal TDP43 WT E-YFP
27450	pRS416 Gal Q331K E-YFP
27449	pRS416Gal-M337V-E-YFP
27448	pRS416-Gal G294A-E-YFP

**Table 2:** Table representing the details of primers used for the qPCR.

Gene Names	Primer Sequences
ALG9	Forward primer: CTTCTGCCGTTGCCATGTTG Reverse primer: GACCCAGTGGACAGATAGCG
CIT3	Forward primer: TTTTGGGTGTTCAAGGGCCA Reverse primer:GCTTCCAGACCCTCCAAGTT
MIH1	Forward primer: TGCAACGGCAAGATGGGAAA Reverse primer: CTGGATGACGCAGACGTGAA
FAA2	Forward primer: CCGGTTACACCAAAGGCTCT Reverse primer: ATGGCAACCGCCTGTTTCTT

## Data Availability

The raw data for yeast transcriptomic has been deposited in the NCBI GEO database (www.ncbi.nlm.nih.gov/bioproject/817798).

## Supplementary Materials

The following supporting information can be downloaded at: https://www.mdpi.com/article/10.3390/cells12091228/s1, Supplementary Data (Figures S1–S6 and Tables S1–S24). Figure S1: Fluorescence images (dark field, bright field and overlay) and bar graph representing results obtained from fluorescence studies of TDP-43 transformed *Saccharomyces cerevisiae*. Figure S2: Scatter plot representing results obtained from flow cytometry analysis of TDP-43 transformed Saccharomyces cerevisiae using Beckman Coulter Flow Cytometer. Percentage of pure E-YFP expressing cells were obtained using FL1 and FL2 LASERS. The results obtained were concordant with the results obtained from fluorescence quantification of microscopic images. Figure S3: Figure representing Principal Component Analysis (PCA) plots for TDP-43 replicates. The red dots represent TDP-43-Wild Type while green dots represent TDP-43 mutants (Top left: TDP-43-Q331K-positive mode, Top right: TDP-43-Q331K negative mode, Bottom left: TDP-43-G294A, Bottom right: TDP-43-M337V). Figure S4: Venn Diagram and table representing common pathways enriched between metabolites obtained from positive and negative mode analysis of TDP-43-Q331K Mutant. Figure S5: Results obtained from GSEA analysis motor neurons from of TDP-43 A315T mutant mice − [(2 Disease Mutants + 2 Controls, Adj.*p*. Value less than 0.05) (KEGG Database)]. Figure S6: Results obtained from GSEA analysis of patient cortex datasets − [(146 Disease + 16 Controls, Adj.*p*. Value less than 0.05) (KEGG Database)]. (Figures were made using www.networkanalyst.ca and Microsoft Excel 2019). Table S1: Significant pathways obtained from pathway enrichment analysis of significant metabolites (TDP-43-Q331K both modes integrated). Table S2: Significant pathways obtained from pathway enrichment analysis of significant metabolites (TDP-43-G294A-Positive Mode). Table S3: Significant pathways obtained from pathway enrichment analysis of significant metabolites (TDP-43-M337V-Positive Mode). Table S4: Yeast mass spectrometry Detected Metabolites (MRM). Table S5: Yeast mass spectrometry (Machine Parameters). Table S6: Yeast mass spectrometry (Solvent Composition). Table S7: Q-PCR melt curve results. Table S8: Volcano plot for differentially expressed significant genes (yeast transcriptomics). Gene Set Enrichment analysis from RNA sequencing of yeast transformed with TDP-43 and its mutant. Table S9: Pathway enrichment analysis from RNA sequencing of yeast transformed with TDP-43 and its mutant. Table S10: Venn diagram representing common pathways enriched between GSEA and Enrichr (Yeast transcriptomics). Table S11: Results obtained from Pathway Enrichment Analysis of metabolomic datasets pertaining to ALS A315T mutant TDP-43 Mice Motor Neuron using KEGG Database. Table S12: Results obtained from Pathway Enrichment Analysis of metabolomic datasets pertaining to ALS Patient CSF using KEGG Database. Table S13: Results obtained from integrated pathway analysis (RNA sequencing and metabolomics) of yeast transformed with TDP-43 and its mutant. Table S14: Bar graph and table representing results obtained from fluorescence studies (Total Cell Florescence) of TDP-43 transformed yeast cells treated with short-chain fatty acids. Table S15: Table representing results of ratios obtained between Total cell fluorescence and aggregate fluorescence for TDP-43 transformed yeast cells treated with short-chain fatty acids. Table S16: Bar graph and table representing results obtained from fluorescence studies (Total Cell Florescence) of TDP-43 transformed yeast cells treated with long-chain fatty acids. Table S17: Table representing results of ratios obtained between Total cell fluorescence and aggregate fluorescence for TDP-43 transformed yeast cells treated with long-chain fatty acids. Table S18: Table representing results obtained from fluorescence quantification (Total Cell Florescence) of TDP-43 transformed yeast cells treated with TCA cycle metabolites and knock-outs. Table S19: Table representing results obtained from fluorescence quantification (Total Cell Florescence) of TDP-43 transformed yeast cells treated with TCA cycle metabolites and knock-outs. Table S20: Table representing results of ratios obtained between Total cell fluorescence and aggregate fluorescence or TDP-43 transformed yeast cells treated with TCA cycle metabolites and knock-outs. Table S21: Bar graph and table representing results obtained from fluorescence quantification (Total Cell Florescence) for TDP-43 transformed yeast cells treated with glutathione and nicotinic acid. Table S22: Quantification results of filter retardation assay carried out on treated Saccharomyces cerevisiae transformed with TDP-43 and its mutant. Table S23: Imaging (dark field, bright field and overlay) and quantification results of metabolite addition experiments show that short-chain fatty acids reduce amyloidogenesis, while long-chain fatty acids increase amyloidogenesis in Saccharomyces cerevisiae transformed with TDP-43 and its mutant. Table S24: Figure summarizing the findings of the study.
