# Supplementary material for: Integrated Omic Analysis Delineates Pathways Modulating Toxic TDP-43 Protein Aggregates in Amyotrophic Lateral Sclerosis
Source: Cells. 2023 Apr 24;12(9):1228. doi: 10.3390/cells12091228 (PMC10177613; doi:10.3390/cells12091228)
Supplement: Supplementary file 1 [file cells-12-01228-s001.zip › cells-2250536-supplementary.pdf]

Integrated Omic analysis delineates pathways modulating toxic TDP-43 protein aggregates in Amyotrophic Lateral Sclerosis

Saiswaroop Rajaratnam[1,#], Akhil P S[1,2,#], Kanikaram Sai Phalguna[1], Sai Sanwid Pradhan[1], Meghana Manjunath[3], Raksha Rao K[3], Rajesh Babu Dandamudi[4], Sai Krishna Srimadh Bhagavatham[1], Sujith Kumar Pulukool[1], Sriram Rathnakumar[1], Sai Kocherlakota[5], Ashish Pargaonkar[6], Ravindra P. Veeranna [7], Natarajan Arumugam[8], Abdulrahman I. Almansour[8], Bibha Choudhary[3,*], Venketesh Sivaramakrishnan[1,*]

[1] Disease Biology Lab, Dept. of Biosciences, Sri Sathya Sai Institute of Higher Learning, Prasanthi Nilayam, Anantapur, Andhra Pradesh, India – 515134.

[2] Central Water and Power Research Station, Khadakwasla, Pune- 411024.

[3] Institute of Bioinformatics and Applied Biotechnology, Bengaluru, Karnataka, India – 560100.

[4] Phenomenex India, Hyderabad, Telangana, India -500084 ;

[5] KU Leuven, Belgium -3000.

[6] Application Division, Agilent Technologies Ltd., Bengaluru, India.

[7] Department of Biochemistry, Council of Scientific & Industrial Research (CSIR) - Central Food Technological Research Institute (CFTRI), Mysuru 570020, Karnataka, India.

[8] Department of Chemistry, College of Science, King Saud University, P.O. Box 2455, Riyadh 11451, Saudi Arabia.

[#]Both these authors contributed equally.

*Author of correspondence

Venketesh Sivaramakrishnan: svenketesh@sssihl.edu.in

Bibha Choudhary: vibha@ibab.ac.in

Key Words: ALS, amyloid, neurodegenerative disease, transcriptomics, metabolomics

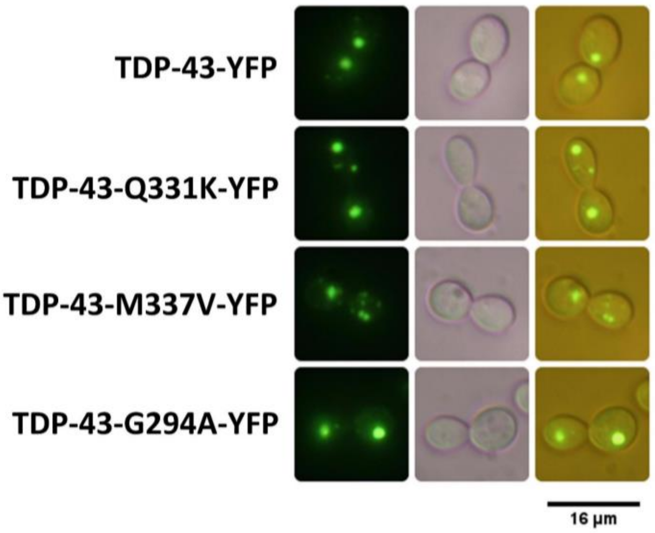

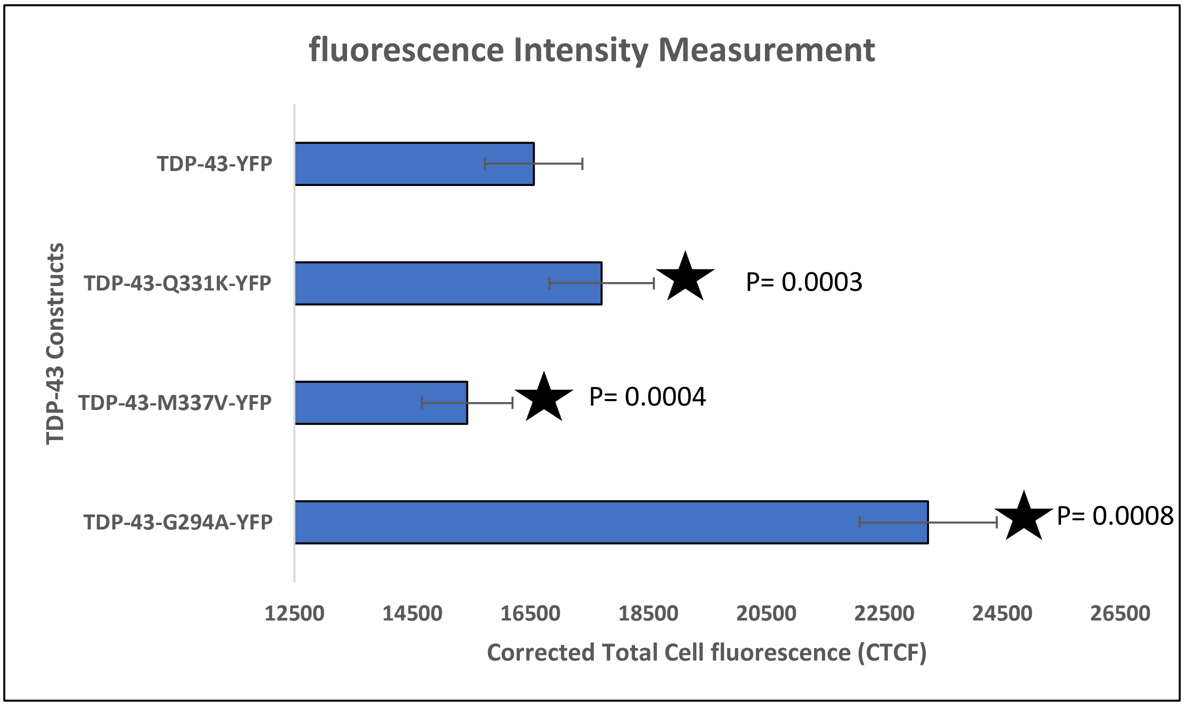

Figure S1: Fluorescence images (dark field, bright field and overlay) and bar graph representing results obtained from fluorescence studies of TDP-43 transformed Saccharomyces cerevisiae.

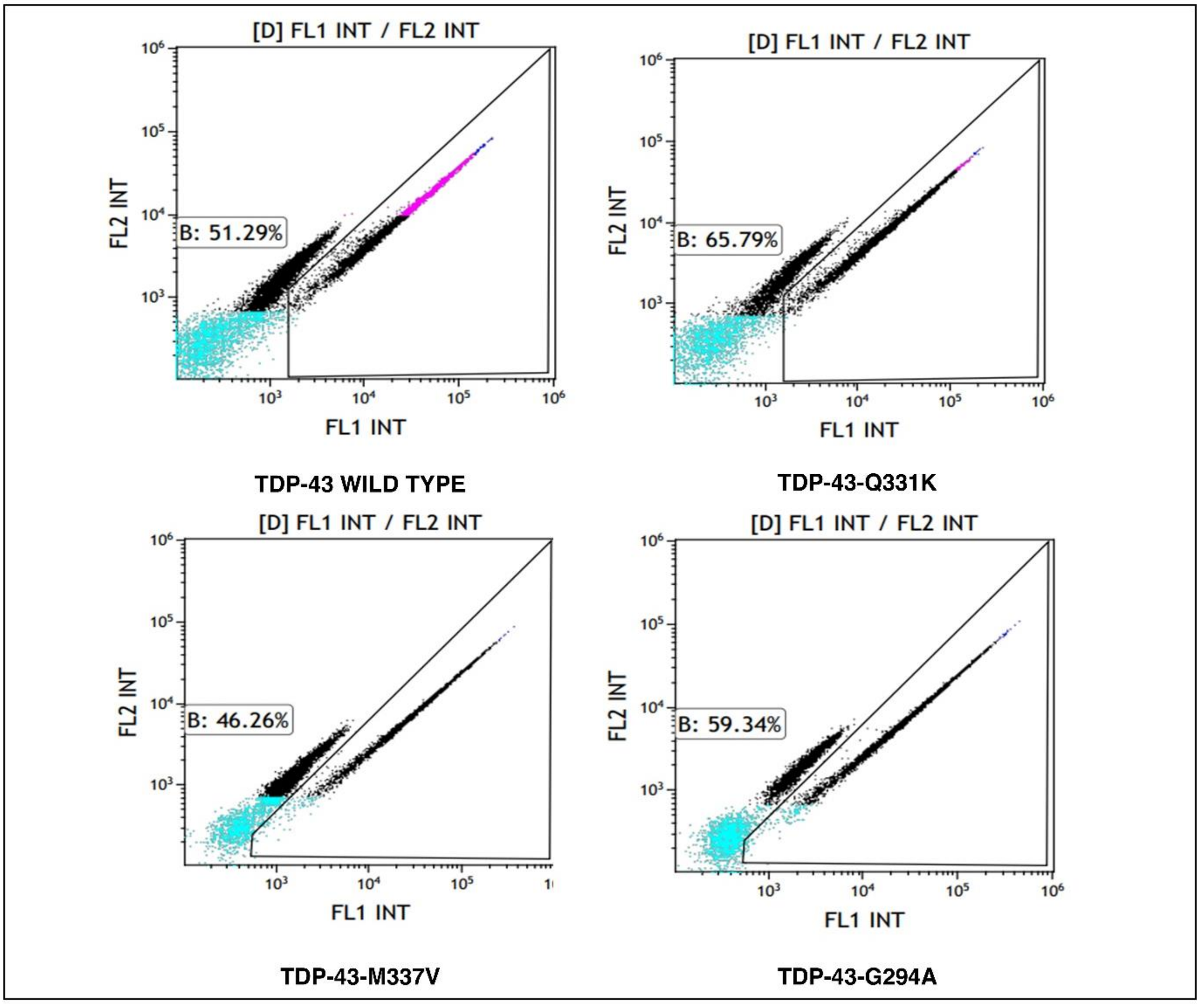

Figure S2: Scatter plot representing results obtained from flow cytometry analysis of TDP-43 transformed Saccharomyces cerevisiae using Beckman Coulter Flow Cytometer. Percentage of pure E-YFP expressing cells were obtained using FL1 and FL2 LASERS. The results obtained were concordant with the results obtained from fluorescence quantification of microscopic images.

PCA PLOT: TDP-43-Q331K-POSITIVE MODE

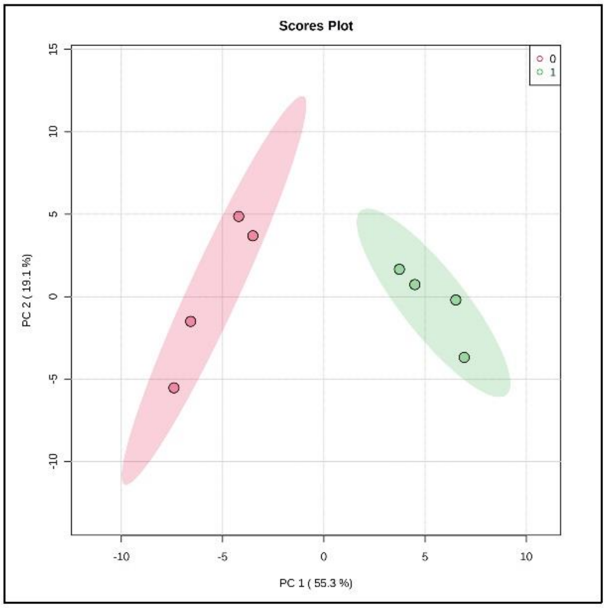

PCA PLOT: TDP-43-Q331K-NEGATIVE MODE

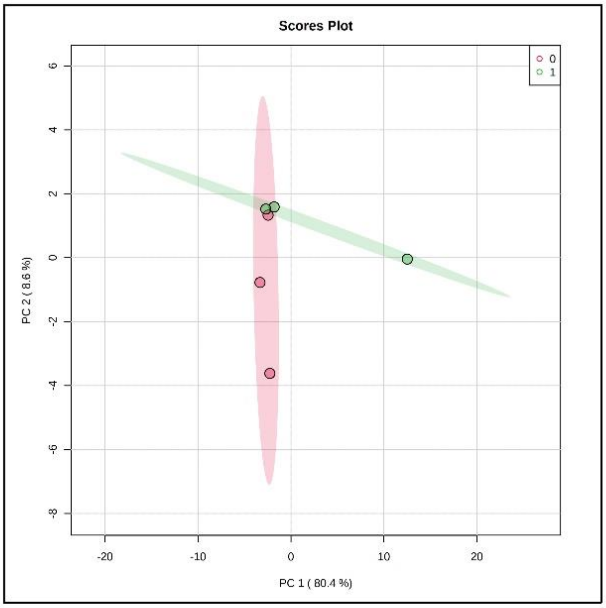

PCA PLOT: TDP-43-G294A-POSITIVE MODE

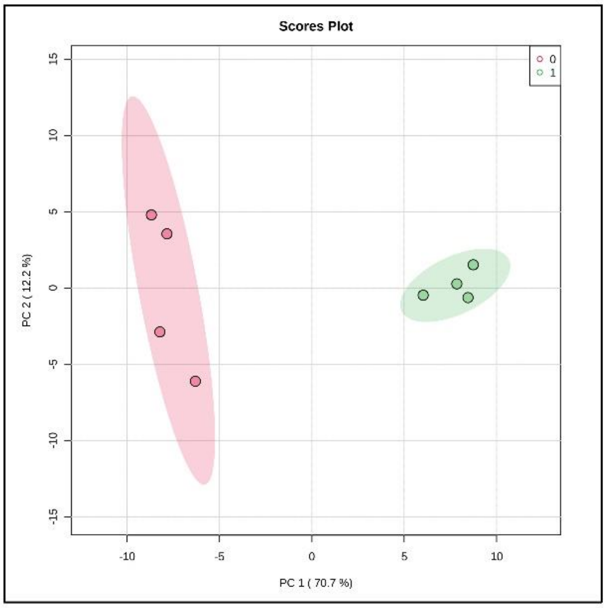

PCA PLOT: TDP-43-M337V-POSITIVE MODE

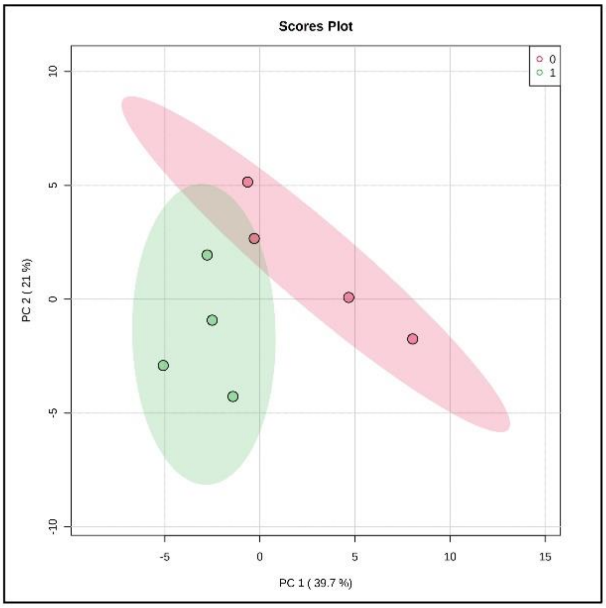

Figure S3: Figure representing Principal Component Analysis (PCA) plots for TDP-43 replicates. The red dots represent TDP-43-Wild Type while green dots represent TDP-43 mutants (Top left: TDP-43-Q331K-positive mode, Top right: TDP-43-Q331K negative mode, Bottom left: TDP-43-G294A, Bottom right: TDP-43-M337V).

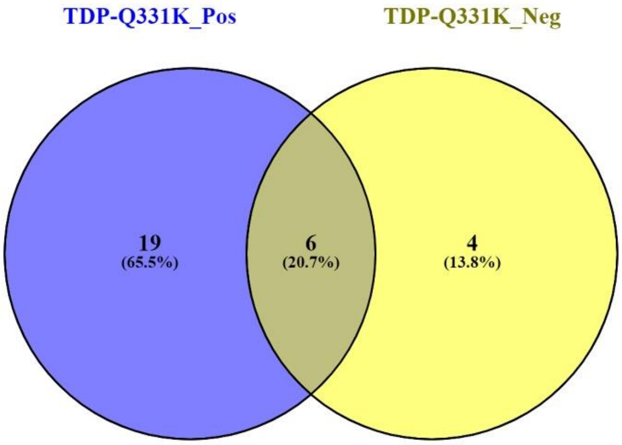

MODES FOR TDP-Q331K	TOTAL	ELEMENTS
TDP-Q331K_Negative mode and TDP-Q331K_Positive mode	6	Alanine, aspartate and glutamate metabolism
		Glyoxylate and dicarboxylate metabolism
		Nicotinate and nicotinamide metabolism
		Purine metabolism
		Sulfur metabolism
		Tyrosine metabolism
TDP-Q331K_Positive mode	19	Pantothenate and CoA biosynthesis
		Porphyrin and chlorophyll metabolism
		Phenylalanine, tyrosine and tryptophan biosynthesis
		Ubiquinone and other terpenoid-quinone biosynthesis
		Lysine biosynthesis
		Glutathione metabolism
		Valine, leucine and isoleucine degradation
		Cyanoamino acid metabolism
		Amino sugar and nucleotide sugar metabolism
		Pyrimidine metabolism
		Tryptophan metabolism
		Methane metabolism
		Cysteine and methionine metabolism
		Thiamine metabolism
		Aminoacyl-tRNA biosynthesis
		Valine, leucine and isoleucine biosynthesis
		Phenylalanine metabolism
		Glycine, serine and threonine metabolism
		Arginine and proline metabolism
TDP-Q331K_Negative mode	4	Citrate cycle (TCA cycle)
		Starch and sucrose metabolism
		Butanoate metabolism
		Propanoate metabolism

Figure S4: Venn Diagram and table representing common pathways enriched between metabolites obtained from positive and negative mode analysis of TDP-43-Q331K Mutant.

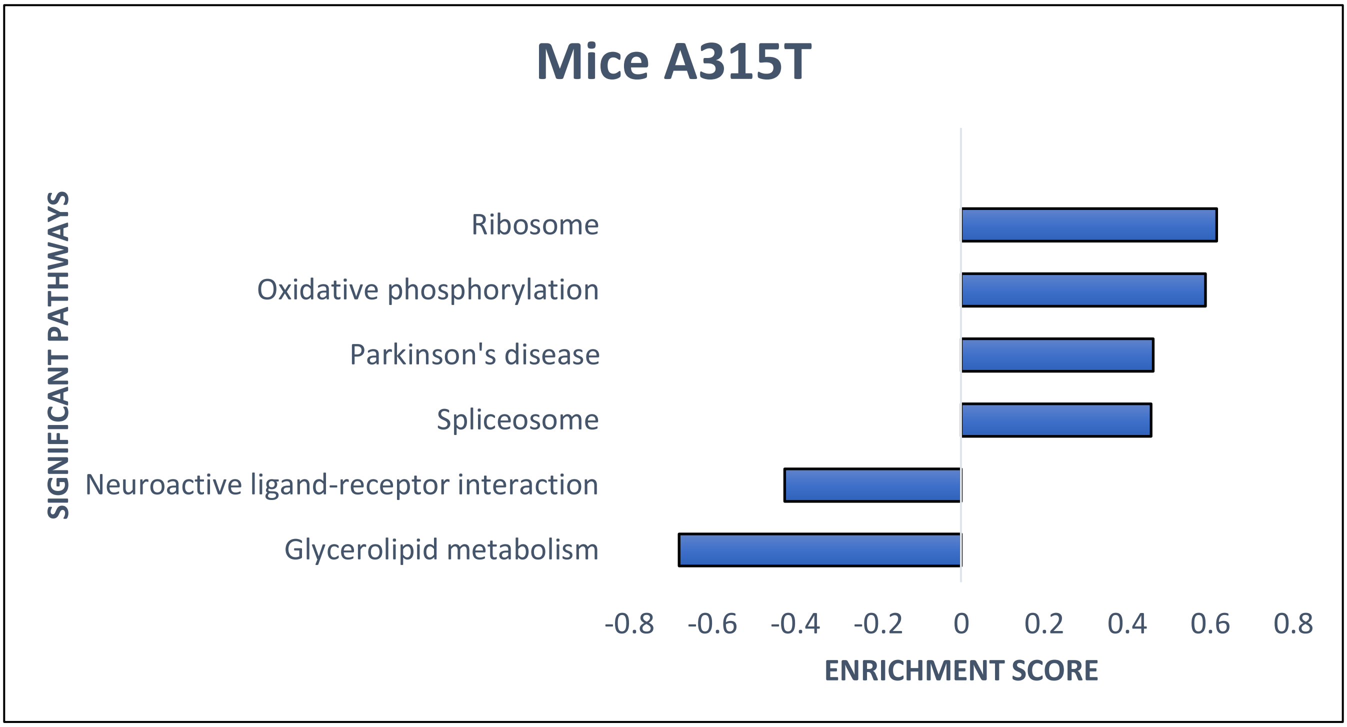

Figure S5: Results obtained from GSEA analysis motor neurons from of TDP-43 A315T mutant mice – [(2 Disease Mutants + 2 Controls, Adj.P. Value less than 0.05) (KEGG Database)].

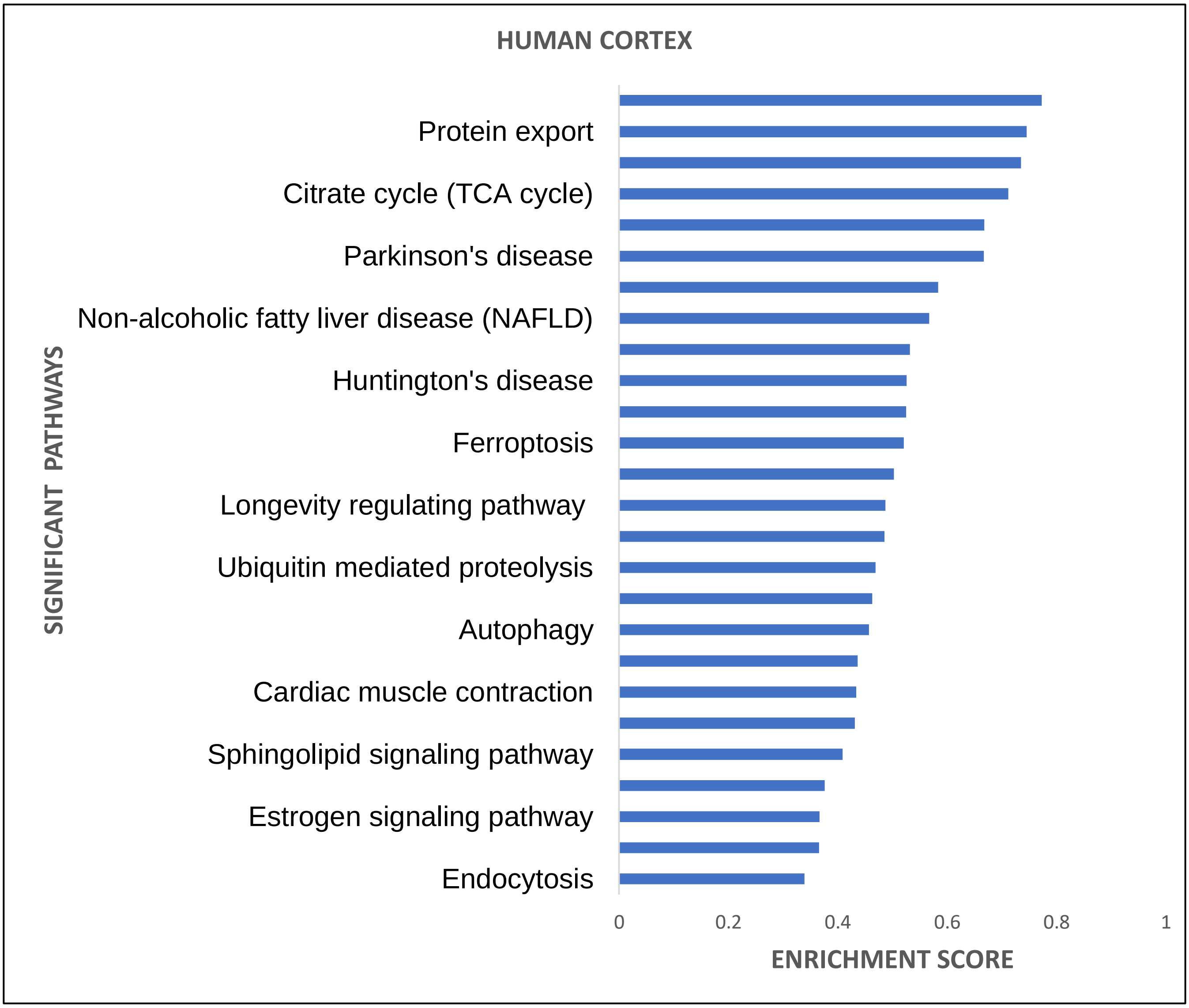

Figure S6: Results obtained from GSEA analysis of patient cortex datasets - [(146 Disease + 16 Controls, Adj.P. Value less than 0.05) (KEGG Database)]. (Figures were made using www.networkanalyst.ca and Microsoft Excel 2019).

PATHWAYS	TOTAL	EXPECTED	HITS	RAW P	FDR	IMPACT
PURINE METABOLISM	152	3.6961	15	3.12E-06	0.00034	0.54237
GLYCINE, SERINE AND THREONINE METABOLISM	81	1.9697	10	2.18E-05	0.001187	0.74074
PEROXISOME	39	0.94835	7	3.41E-05	0.001238	0
RIBOSOME	181	4.4013	12	0.001361	0.037099	0
PYRIMIDINE METABOLISM	94	2.2858	8	0.001881	0.040997	0.4537
THIAMINE METABOLISM	50	1.2158	5	0.006919	0.1257	0.37778
VALINE, LEUCINE AND ISOLEUCINE BIOSYNTHESIS	35	0.85109	4	0.00973	0.15152	0.66667
GLYOXYLATE AND DICARBOXYLATE METABOLISM	92	2.2371	6	0.023811	0.32443	0.28916
RNA POLYMERASE	30	0.7295	3	0.035329	0.38675	0
TRYPTOPHAN METABOLISM	101	2.456	6	0.035537	0.38675	0.2381
CYANOAMINO ACID METABOLISM	53	1.2888	4	0.039029	0.38675	0.041667
CYSTEINE AND METHIONINE METABOLISM	106	2.5776	6	0.043425	0.39445	0.31532
ALANINE, ASPARTATE AND GLUTAMATE METABOLISM	60	1.459	4	0.057193	0.42286	0.44231
MAPK SIGNALING PATHWAY - YEAST	114	2.7721	6	0.058185	0.42286	0.09375
NUCLEOTIDE EXCISION REPAIR	37	0.89972	3	0.059884	0.42286	0
ABC TRANSPORTERS	145	3.5259	7	0.062071	0.42286	0
PROPANOATE METABOLISM	63	1.532	4	0.066153	0.42416	0.26667
PANTOTHENATE AND COA BIOSYNTHESIS	44	1.0699	3	0.090504	0.54805	0.30233
SULFUR METABOLISM	48	1.1672	3	0.1104	0.63334	0.12903
PHENYLALANINE, TYROSINE AND TRYPTOPHAN BIOSYNTHESIS	52	1.2645	3	0.13181	0.71835	0.31667
HIPPO SIGNALING PATHWAY - MULTIPLE SPECIES	8	0.19453	1	0.17887	0.92844	0.090909

Table S1: Significant pathways obtained from pathway enrichment analysis of significant metabolites (TDP-43-Q331K both modes integrated).

PATHWAYS	TOTAL	EXPECTED	HITS	RAW P	FDR	IMPACT
AMINOACYL-TRNA BIOSYNTHESIS	46	2.6599	13	4.22E-07	3.08E-05	0
ARGININE BIOSYNTHESIS	18	1.0408	5	0.002564	0.093567	0.36182
ARGININE AND PROLINE METABOLISM	25	1.4456	5	0.011654	0.28358	0.28571
NITROGEN METABOLISM	5	0.28912	2	0.02931	0.5349	0
GLUTATHIONE METABOLISM	26	1.5034	4	0.057604	0.83835	0.11547
CYANOAMINO ACID METABOLISM	8	0.46259	2	0.073401	0.83835	0
VALINE, LEUCINE AND ISOLEUCINE DEGRADATION	18	1.0408	3	0.080389	0.83835	0
GLYCINE, SERINE AND THREONINE METABOLISM	32	1.8503	4	0.10743	0.8476	0.27463
UBIQUINONE AND OTHER TERPENOID-QUINONE BIOSYNTHESIS	2	0.11565	1	0.11236	0.8476	0
PHENYLALANINE, TYROSINE AND TRYPTOPHAN BIOSYNTHESIS	21	1.2143	3	0.11611	0.8476	0.02144
ALANINE, ASPARTATE AND GLUTAMATE METABOLISM	22	1.2721	3	0.12916	0.85717	0.51798
NICOTINATE AND NICOTINAMIDE METABOLISM	12	0.69388	2	0.14944	0.90908	0.19876
CARBAPENEM BIOSYNTHESIS	3	0.17347	1	0.16381	0.91984	0
GLYOXYLATE AND DICARBOXYLATE METABOLISM	26	1.5034	3	0.18599	0.9698	0.10442

Table S2 : Significant pathways obtained from pathway enrichment analysis of significant metabolites (TDP-43-G294A-Positive Mode).

PATHWAYS	TOTAL	EXPECTED	HITS	RAW P	FDR	IMPACT
PURINE METABOLISM	62	1.4059	5	0.009942	0.68502	0.10325
TRYPTOPHAN METABOLISM	30	0.68027	3	0.027391	0.68502	0.36047
NICOTINATE AND NICOTINAMIDE METABOLISM	12	0.27211	2	0.028151	0.68502	0.19876
GLYCINE, SERINE AND THREONINE METABOLISM	32	0.72562	2	0.16162	1	0
PYRIMIDINE METABOLISM	34	0.77098	2	0.178	1	0.01998
CYSTEINE AND METHIONINE METABOLISM	41	0.92971	2	0.23719	1	0.1717
PHENYLALANINE, TYROSINE AND TRYPTOPHAN BIOSYNTHESIS	21	0.47619	1	0.38571	1	0
AMINOACYL-TRNA BIOSYNTHESIS	46	1.0431	1	0.66152	1	0

Table S3 : Significant pathways obtained from pathway enrichment analysis of significant metabolites (TDP-43-M337V-Positive Mode).

Table S4 : Yeast mass spectrometry Detected Metabolites (MRM).

Compound Name	ISD	Precursor ion	Product ion	Dwell	Frag(V)	CE(V)	Polarity
(L)-arginino-succinate	FALSE	291.1299	70	3	380	37	POSITIVE
2-Aminobutyraldehyde	FALSE	88.0757	70.1	3	380	9	POSITIVE
3-phospho-serine	FALSE	186.0162	88	3	380	12	POSITIVE
4 Acetamidobutanoic acid	FALSE	146.08	86.1	3	380	9	POSITIVE
4 Hydroxybutyric acid	FALSE	105.0546	45	3	380	5	POSITIVE
4-Coumarate	FALSE	165.0446	119	3	380	10	POSITIVE
5-Hydroxyindoleacetic acid	FALSE	192.0655	146	3	380	10	POSITIVE
5-oxo-proline	FALSE	130.0499	84	3	380	10	POSITIVE
Acetyl Carnitine	FALSE	204.123	85	3	380	9	POSITIVE
Allo-Threonine	FALSE	120.0655	74.1	3	380	5	POSITIVE
Aminobutyric acid	FALSE	104.0706	44.1	3	380	9	POSITIVE
Arginine	FALSE	175.119	70.1	3	380	25	POSITIVE
betaine aldehyde	FALSE	103.0992	58	3	380	21	POSITIVE
Carnitine	FALSE	162.1125	60.1	3	380	12	POSITIVE
citrulline	FALSE	176.103	159	3	380	5	POSITIVE
Creatinine	FALSE	114.0662	44	3	380	17	POSITIVE
Cytidine 5 -Monophosphate (5-CMP)	FALSE	324	95	3	380	40	POSITIVE
Cytosine	FALSE	112.1	95.1	3	380	17	POSITIVE
DL-Pipecolic acid	FALSE	130.0863	84	3	380	18	POSITIVE
Erythrono-1,4-lactone	FALSE	119.11	91.1	3	380	10	POSITIVE
Ethylmalonate/Methyl Malonate	FALSE	133.0495	115.04	3	380	10	POSITIVE
Glucosamine/Galactosamine	FALSE	180.0866	162	3	380	5	POSITIVE
Glutamic acid	FALSE	148.0604	84	3	380	17	POSITIVE
Glycine	FALSE	76.0393	30	3	380	9	POSITIVE
Gly-Gly	FALSE	133.0608	30	3	380	20	POSITIVE
Gly-Leu	FALSE	189.1234	130	3	380	10	POSITIVE
Homoserine	FALSE	120.0655	74.1	3	380	9	POSITIVE
Hypoxanthine	FALSE	137	119	3	380	21	POSITIVE
Isoleucine	FALSE	132.1019	86.1	3	380	9	POSITIVE
Kynurenic acid	FALSE	190.0499	144.1	3	380	16	POSITIVE
Kynurenine	FALSE	209.0921	192	3	380	5	POSITIVE
L-anthranilic acid	FALSE	139	121	3	380	12	POSITIVE
L-Arginine	FALSE	177.1	159.1	3	380	10	POSITIVE
L-Thymine	FALSE	131.1	113.9	3	380	15	POSITIVE
L-Tryptophan	TRUE	207.1	189	3	380	10	POSITIVE
Lysine	FALSE	147.1128	130.2	3	380	5	POSITIVE
Methyl Nicotinamide	FALSE	138.08	95.1	3	380	25	POSITIVE
Methylglutaric acid	FALSE	147.0652	55.05423	3	380	10	POSITIVE
Myristoleic acid	FALSE	227.2006	43.1	3	380	25	POSITIVE
N,N Dimethyl Glycine/Methyl alanine	FALSE	104.0706	58.1	3	380	9	POSITIVE

N-acetylalanine	FALSE	132.0655	44.1	3	380	20	POSITIVE
N-Acetylneuraminic acid	FALSE	310.1	121	3	380	21	POSITIVE
N-Acetylornithine	FALSE	175.1077	70.1	3	380	29	POSITIVE
N-carbamoyl-L-aspartate	FALSE	177.0506	74	3	380	19	POSITIVE
Nicotinamide	FALSE	123.0553	80.1	3	380	20	POSITIVE
Nonadecanoic acid	FALSE	298.2866	281.4	3	380	10	POSITIVE
Octonyl Carnitine	FALSE	288.2	85	3	380	21	POSITIVE
Oleic acid	FALSE	283.27	89.2	3	380	13	POSITIVE
Phenyl alanine	FALSE	166.0963	120.1	3	380	13	POSITIVE
Proline	FALSE	116.0706	70.1	3	380	17	POSITIVE
Putrescine	FALSE	89.1073	72.1	3	380	8	POSITIVE
SAH	FALSE	385.1289	136.1	3	380	33	POSITIVE
Serine	FALSE	106.0499	60.1	3	380	9	POSITIVE
Thymine-d4	FALSE	131	113.9	3	380	16	POSITIVE
Tryptophan	FALSE	205.1	146.1	3	380	10	POSITIVE
Tyrosine	FALSE	182.0812	136.1	3	380	9	POSITIVE
Uric acid	FALSE	169	141	3	380	13	POSITIVE
Valine	FALSE	118.0863	72.2	3	380	9	POSITIVE
Xanthine	FALSE	153.04	110	3	380	21	POSITIVE
4-Methyl-2-oxo pentanoic acid	FALSE	131.0703	71	3	380	10	POSITIVE
Adenine	FALSE	136.6	119	3	380	10	POSITIVE
AMP	FALSE	348.0704	136	3	380	17	POSITIVE
Asparagine	FALSE	133.0608	74	3	380	13	POSITIVE
Cystathionine	FALSE	223.0747	134.1	3	380	8	POSITIVE
dGMP	FALSE	348.0704	135	3	380	38	POSITIVE
Glutamine	FALSE	147.0764	130.1	3	380	5	POSITIVE
Glu-Tyr	FALSE	311.1238	293.1	3	380	10	POSITIVE
Glycerol 3-phosphate	FALSE	173.02	132	3	380	5	POSITIVE
Histamine	FALSE	112.0869	95.1	3	380	13	POSITIVE
Histidine	FALSE	156.0768	110.1	3	380	9	POSITIVE
Hydroxy Stearic acid Methy ester	FALSE	315.3	97.1	3	380	9	POSITIVE
Isovaleryl Carnitine	FALSE	246.2	85	3	380	17	POSITIVE
Myo-Inositol	FALSE	181.07	139.9	3	380	5	POSITIVE
N-acetyl Glucosamine 6 Phos	FALSE	302.0635	284.1	3	380	5	POSITIVE
Nicotinamide ribotide	FALSE	335	123	3	380	30	POSITIVE
Quinic acid	FALSE	193.0707	111.04	3	380	10	POSITIVE
thiamine	FALSE	266.1196	122	3	380	19	POSITIVE
Uracil	FALSE	113	72	3	380	4	POSITIVE
1,3-diphopshateglycerate	FALSE	265	79	3	380	37	NEGATIVE
2,3-dihydroxybenzoic acid	FALSE	153	109	3	380	19	NEGATIVE
6-phospho-D-gluconate	FALSE	275	97	3	380	13	NEGATIVE

ADP	FALSE	426.1	159	3	380	27	NEGATIVE
anthranilate	FALSE	136	92	3	380	18	NEGATIVE
GDP	FALSE	426.12	159	3	380	27	NEGATIVE
dihydroorotate	FALSE	157	113	3	380	14	NEGATIVE
D-sedoheptulose-1-7-phosphate	FALSE	289	97	3	380	27	NEGATIVE
Eicasatetraenoic acid-D8 (ETA)	FALSE	311.5	59.05	3	380	35	NEGATIVE
Farnesyl-PP	FALSE	381	78.7	3	380	40	NEGATIVE
Frctose-6-Phosphate	FALSE	259	79.1	3	380	37	NEGATIVE
Fumarate	FALSE	119	74	3	380	5	NEGATIVE
Fumaric acid	FALSE	115	71.1	3	380	5	NEGATIVE
Glucose-6-Phosphate	FALSE	259	97	3	380	5	NEGATIVE
Glucuronic acid	FALSE	193	73.1	3	380	9	NEGATIVE
Glutamine	FALSE	147	111	3	380	9	NEGATIVE
hexose-phosphate	FALSE	259	79	3	380	42	NEGATIVE
Hydroxyisocaproic acid	FALSE	131.006	85.1	3	380	16	NEGATIVE
inosine	FALSE	267	135	3	380	27	NEGATIVE
Ketoglutarate	FALSE	145	101.1	3	380	5	NEGATIVE
lactate	FALSE	89	43.2	3	380	16	NEGATIVE
L-Anthranilic aicd	FALSE	137	93.1	3	380	15	NEGATIVE
L-Glutamic acid D5	FALSE	151	107	3	380	13	NEGATIVE
L-Jasmonic acid	TRUE	209	59	3	380	10	NEGATIVE
L-Tryptophan	TRUE	205	117	3	380	10	NEGATIVE
L-Zeatine	TRUE	218	133.5	3	380	21	NEGATIVE
Malic acid	FALSE	133	115.1	3	380	5	NEGATIVE
Maltotetraose	FALSE	665.2	161.1	3	380	13	NEGATIVE

N-acety AA-d3	FALSE	177	90.8	3	380	6	NEGATIV E
N-Aetyl Aspartic acid	FALSE	174	88.1	3	380	13	NEGATIV E
octulose-monophosphate (O8P-O1P)	FALSE	319	97	3	380	22	NEGATIV E
p-aminobenzoate	FALSE	136.05	92	3	380	18	NEGATIV E
PEP	FALSE	167	79	3	380	9	NEGATIV E
Pyruvate	FALSE	89	44	3	380	10	NEGATIV E
Ribose-5-phosphate	FALSE	229.01	96.97	3	380	15	NEGATIV E
shikimate	FALSE	173	93	3	380	20	NEGATIV E
sn-glycerol-3-phosphate	FALSE	171	79	3	380	15	NEGATIV E
S-ribosyl-L-homocysteine_neg	FALSE	266	134	3	380	20	NEGATIV E
succinate	FALSE	117	73	3	380	12	NEGATIV E
taurine	FALSE	124	80	3	380	18	NEGATIV E
Tetradecanoic acid-D3 (TDA-D3)	FALSE	230.3	58	3	380	40	NEGATIV E
trehalose-6-Phosphate	FALSE	421	79	3	380	36	NEGATIV E
UDP-D-glucose	FALSE	565	323	3	380	25	NEGATIV E
UDP-N-acetyl-glucosamine	FALSE	606	385	3	380	28	NEGATIV E
Uric acid	FALSE	167.001	124	3	380	17	NEGATIV E
uridine	FALSE	243	200	3	380	21	NEGATIV E
Zeatine	FALSE	218	173	3	380	10	NEGATIV E

Table S5 : Yeast mass spectrometry (Machine Parameters).

**Source Parameters**		
Parameter	Value (+)	Value (-)
Gas Temp (°C)	250	250
Gas Flow (l/min)	14	14
Nebulizer (psi)	20	20
SheathGasHeater	350	350
SheathGasFlow	12	12
Capillary (V)	3000	3000
VCharging	1000	1000

**Ion Funnel Parameters**	
Pos High Pressure RF	150
Pos Low Pressure RF	60
Neg High Pressure RF	150
Neg Low Pressure RF	60

**Auxiliary**	
Draw Speed	100.0 µL/min
Eject Speed	100.0 µL/min
Draw Position Offset	0.0 mm
Wait Time After Drawing	2.0 s
Vial/Well bottom sensing	Yes

**Injection**	
Injection Mode	Injection with needle wash
Injection Volume	2.00 µL

**Needle Wash**	
Needle Wash Location	Flush Port
Wash Time	10.0 s
Flow	0.300 mL/min
Use Solvent Types	Yes
Stroke Mode	Synchronized
Low Pressure Limit	0.00 bar
High Pressure Limit	600.00 bar
Max. Flow Ramp Up	100.000 mL/min^2
Max. Flow Ramp Down	100.000 mL/min^2
Expected Mixer	No check

**Stop Time**	
Stoptime Mode	Time set
Stoptime	23.00 min
Post time mode	Time set
Post time	5.00min

Table S6: Yeast mass spectrometry (Solvent Composition).

Column Mode	Solvent A	Solvent B
Water's Positive Mode	Grade water (Water, Optima™ LC/MS Grade, Cat. No. W6500, Fisher Chemical™, Fair Lawn, NJ, USA) + 0.1% formic acid (FA) (Formic Acid, 99.0 + %, Optima LC/MS grade, Cat. No. A117-50, Fisher Chemical, Fisher Scientific, Fair Lawn, NJ, USA)	100% Acetonitrile (ACN) (Acetonitrile, Optima LC/MS grade, Cat. No. A955, Fisher Chemical, Fisher Scientific, Fair Lawn, NJ, USA) + 0.1% formic acid (FA)
Water's Negative Mode	20 mM Ammonium acetate (Ammonium Acetate (Optima LC/MS), Cat. No. A11450 Fisher Chemical, Fisher Scientific, Fair Lawn, NJ, USA) pH 9 in water	20 mM Ammonium acetate (Ammonium Acetate (Optima LC/MS), Cat. No. A11450 Fisher Chemical, Fisher Scientific, Fair Lawn, NJ, USA) pH 9 in Acetonitrile

Timetable			
Time	Function	Parameter	Solvent_composition
1	3.00 min	Change Solvent Composition	Solvent Composition A:70.00% B:30.00 %
2	3.00 min	Change Flow	Flow: 0.3 mL/min
3	12.00 min	Change Solvent Composition	Solvent Composition A:98.00% B:2.00 %
4	12.00 min	Change Flow	Flow: 0.3 mL/min
5	15.00 min	Change Solvent Composition	Solvent Composition A:98.00% B:2.00 %
6	15.00 min	Change Flow	Flow: 0.3 mL/min
7	16.00 min	Change Solvent Composition	Solvent Composition A:15.00% B:85.00 %
8	16.00 min	Change Flow	Flow: 0.3 mL/min
9	23.00 min	Change Solvent Composition	Solvent Composition A:15.00% B:85.00 %
10	23.00 min	Change Flow	Flow: 0.3 mL/min

Table S7: Q-PCR melt curve results.

Well	Target Name	Tm	Melt Peak Height
1	SC_ctrl_ALG9	78.472	33,664.000
2	SC_ctrl_ALG9	78.472	35,361.316
3	SC_ctrl_ALG9	78.359	30,643.055
4	SC_ctrl_ALG9	78.472	31,334.941
5	SC_mut_ALG9	78.359	32,732.633
6	SC_mut_ALG9	78.359	40,384.113
7	SC_mut_ALG9	78.472	35,623.656
8	SC_mut_ALG9	78.359	33,932.109
9	SC_ctrl_CIT3	80.511	36,493.750
10	SC_ctrl_CIT3	80.624	25,483.025
11	SC_ctrl_CIT3	80.511	28,394.873
12	SC_ctrl_CIT3	80.964	8,798.745
13	SC_mut_CIT3	80.511	28,166.035
14	SC_mut_CIT3	80.511	21,052.998
15	SC_mut_CIT3	80.511	25,464.791
16	SC_mut_CIT3	80.511	24,980.783
17	SC_ctrl_MIH1	77.113	68,102.141
18	SC_ctrl_MIH1	76.999	61,921.539
19	SC_ctrl_MIH1	76.999	51,552.285
20	SC_ctrl_MIH1	77.113	55,044.328
21	Sc_mut_MIH1	76.773	36,258.695
22	Sc_mut_MIH1	76.659	30,236.443
23	Sc_mut_MIH1	76.773	29,325.252
24	Sc_mut_MIH1	76.546	23,707.949
25	Sc_ctrl_FAA2	77.226	34,361.215
26	Sc_ctrl_FAA2	77.226	31,186.855
27	Sc_ctrl_FAA2	77.226	23,374.393
28	SC_mut_FAA2	77.226	22,494.443
29	SC_mut_FAA2	77.226	29,181.404
30	SC_mut_FAA2	77.113	19,344.182
31	SC_mut_FAA2	77.226	26,075.854

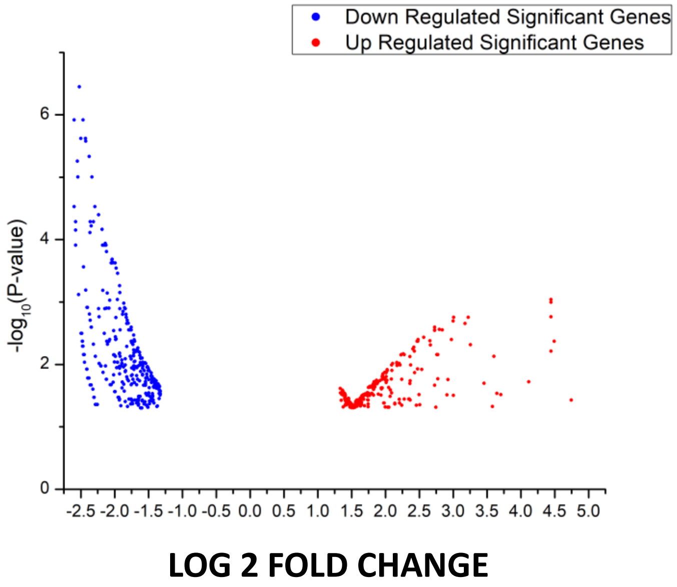

	Name	Total	Hits	Enrichment Score	Pval	Padj
1	Oxidative phosphorylation	72	42	0.409775486	0.045752	0.544538
2	Ubiquitin mediated proteolysis	47	33	0.462418922	0.046668	0.544538
3	Peroxisome	39	28	0.460739276	0.05137	0.544538
4	RNA degradation	62	42	0.423299147	0.060732	0.544538
5	Basal transcription factors	32	23	0.468586572	0.06281	0.544538
6	N-Glycan biosynthesis	31	21	0.476105686	0.065546	0.544538
7	Cysteine and methionine metabolism	40	26	-0.432035361	0.072864	0.544538
8	Various types of N-glycan biosynthesis	30	21	0.464906245	0.080672	0.544538
9	RNA polymerase	30	21	-0.438799753	0.110565	0.663391
10	Glycine, serine and threonine metabolism	31	27	-0.37219147	0.175309	0.879288
11	Protein processing in endoplasmic reticulum	88	67	0.324227886	0.1792	0.879288
12	Proteasome	35	25	0.3786746	0.224832	0.879288
13	Nucleotide excision repair	37	27	0.377207882	0.227806	0.879288
14	Biosynthesis of secondary metabolites	300	199	-0.224054013	0.251012	0.879288
15	MAPK signaling pathway - yeast	114	78	0.291927513	0.256881	0.879288
16	Biosynthesis of antibiotics	229	153	-0.23132219	0.263538	0.879288
17	Methane metabolism	26	20	0.390863235	0.293919	0.879288
18	Citrate cycle (TCA cycle)	32	20	-0.35393291	0.309756	0.879288
19	Homologous recombination	20	16	0.397133247	0.336222	0.879288
20	Ribosome	183	123	0.255747186	0.340087	0.879288

Table S8 : Volcano plot for differentially expressed significant genes (yeast transcriptomics). Gene Set Enrichment analysis from RNA sequencing of yeast transformed with TDP-43 and its mutant.

Term	P-value	Adjusted P-value	Genes
Ribosome	8.42209E-14	4.46371E-12	RPL23A;RPL22A;RPS4A;RPL22B;RPL33B;RPL20A;RPL20B;RPL6A;RPS23A;RPL8B;RPS14B;RPS16B;RPS26A;RPL16A;RPS11B;RPS9B;RPL8A
Valine, leucine and isoleucine biosynthesis	0.000123292	0.002178166	ILV3;BAT1;LEU9
Glyoxylate and dicarboxylate metabolism	0.000113579	0.002178166	FDH1;GCV2;CIT3;CTA1
Peroxisome	0.000321299	0.004257216	DCI1;FAA2;POX1;CTA1
ABC transporters	0.001449674	0.011983393	PDR5;SNQ2
MAPK signaling pathway	0.000418369	0.004434716	PTP2;MKC7;RLM1;SST2;CTA1;MCM1
Pyrimidine metabolism	0.0016624	0.011983393	URA7;URA2;FUR1
Glycine, serine and threonine metabolism	0.002034916	0.011983393	THR4;SER2;GCV2
RNA polymerase	0.002034916	0.011983393	RPA49;RPC19;RPA43
Pantothenate and CoA biosynthesis	0.006933521	0.036747662	ILV3;BAT1
Thiamine metabolism	0.011044769	0.048781064	PHO12;THI80
Fatty acid degradation	0.011044769	0.048781064	FAA2;POX1
Methane metabolism	0.018746714	0.076428912	SER2;FDH1
Alanine, aspartate and glutamate metabolism	0.028116413	0.093135617	URA2;ASN1
Meiosis	0.024587792	0.090778446	PDS1;HXT3;SNF3;REC8
Cysteine and methionine metabolism	0.040912737	0.12046528	BAT1;SAM4
Ribosome biogenesis in eukaryotes	0.025692013	0.090778446	RCL1;UTP10;UTP21

Table S9: Pathway enrichment analysis from RNA sequencing of yeast transformed with TDP-43 and its mutant.

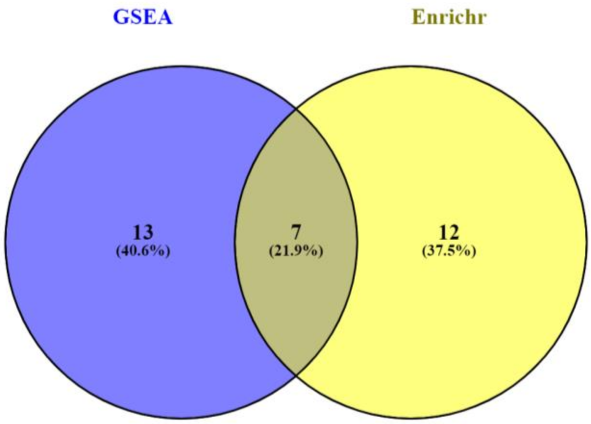

Names	Total	Elements
Enrichr and GSEA	7	RNA POLYMERASE
		METHANE METABOLISM
		PEROXISOME
		RIBOSOME
		NUCLEOTIDE EXCISION REPAIR
		CYSTEINE AND METHIONINE METABOLISM
		GLYCINE, SERINE AND THREONINE METABOLISM
GSEA	13	PROTEASOME
		OXIDATIVE PHOSPHORYLATION
		BIOSYNTHESIS OF ANTIBIOTICS
		VARIOUS TYPES OF N-GLYCAN BIOSYNTHESIS
		HOMOLOGOUS RECOMBINATION
		BASAL TRANSCRIPTION FACTORS
		CITRATE CYCLE (TCA CYCLE)
		RNA DEGRADATION
		MAPK SIGNALING PATHWAY - YEAST
		BIOSYNTHESIS OF SECONDARY METABOLITES
		UBIQUITIN MEDIATED PROTEOLYSIS
		N-GLYCAN BIOSYNTHESIS
		PROTEIN PROCESSING IN ENDOPLASMIC RETICULUM
Enrichr	12	HIPPO SIGNALING PATHWAY
		MEIOSIS
		PYRIMIDINE METABOLISM
		VALINE, LEUCINE AND ISOLEUCINE BIOSYNTHESIS
		ALANINE, ASPARTATE AND GLUTAMATE METABOLISM
		ABC TRANSPORTERS
		GLYOXYLATE AND DICARBOXYLATE METABOLISM
		MAPK SIGNALING PATHWAY
		PANTOTHENATE AND COA BIOSYNTHESIS
		FATTY ACID DEGRADATION
		THIAMINE METABOLISM
		RIBOSOME BIOGENESIS IN EUKARYOTES

Table S10: Venn diagram representing common pathways enriched between GSEA and Enrichr (Yeast transcriptomics).

Pathways	Total	Expected	Hits	Raw p	(-LOG10(p))	Holm adjust	FDR	Impact
Glycine, serine and threonine metabolism	34	1.0385	7	4.49E-05	4.3477	0.0037718	0.0037718	0.38273
Citrate cycle (TCA cycle)	20	0.61089	5	0.00023468	3.6295	0.019478	0.0098564	0.24531
Glyoxylate and dicarboxylate metabolism	32	0.97742	5	0.0023142	2.6356	0.18976	0.064796	0.24075
Alanine, aspartate and glutamate metabolism	28	0.85525	4	0.0091196	2.04	0.73869	0.15321	0.04808
Glutathione metabolism	28	0.85525	4	0.0091196	2.04	0.73869	0.15321	0.35188
Purine metabolism	66	2.0159	6	0.013232	1.8784	1	0.18524	0.17015
beta-Alanine metabolism	21	0.64143	3	0.024129	1.6175	1	0.28718	0.05597
Pyruvate metabolism	22	0.67198	3	0.027351	1.563	1	0.28718	0.23794
Glycolysis / Gluconeogenesis	26	0.79416	3	0.042418	1.3724	1	0.3959	0.20615
Arginine biosynthesis	14	0.42762	2	0.065791	1.1818	1	0.55265	0.20305
Cysteine and methionine metabolism	33	1.008	3	0.0768	1.1146	1	0.58647	0.05983
Arginine and proline metabolism	38	1.1607	3	0.10695	0.9708	1	0.74868	0.03401
Riboflavin metabolism	4	0.12218	1	0.1168	0.93254	1	0.75474	0
Pentose phosphate pathway	22	0.67198	2	0.1434	0.84344	1	0.84045	0
Propanoate metabolism	23	0.70252	2	0.15413	0.81212	1	0.84045	0.04061
D-Glutamine and D-glutamate metabolism	6	0.18327	1	0.17009	0.76932	1	0.84045	0
Nitrogen metabolism	6	0.18327	1	0.17009	0.76932	1	0.84045	0
Thiamine metabolism	7	0.21381	1	0.19554	0.70876	1	0.91253	0
Valine, leucine and isoleucine biosynthesis	8	0.24436	1	0.22023	0.65713	1	0.97364	0
Biotin metabolism	10	0.30544	1	0.2674	0.57284	1	1	0
Glycerophospholipid metabolism	36	1.0996	2	0.30167	0.52047	1	1	0.00937
Pyrimidine metabolism	39	1.1912	2	0.33602	0.47363	1	1	0.06137
Tryptophan metabolism	41	1.2523	2	0.35869	0.44528	1	1	0.14484
Tyrosine metabolism	42	1.2829	2	0.36993	0.43188	1	1	0.11085
Butanoate metabolism	15	0.45817	1	0.37345	0.42777	1	1	0
Nicotinate and nicotinamide metabolism	15	0.45817	1	0.37345	0.42777	1	1	0.1943
Glycerolipid metabolism	16	0.48871	1	0.39278	0.40585	1	1	0.09346
Aminoacyl-tRNA biosynthesis	48	1.4661	2	0.43565	0.36087	1	1	0
Pantothenate and CoA biosynthesis	19	0.58035	1	0.44733	0.34937	1	1	0.00714
Lysine degradation	25	0.76361	1	0.54245	0.26564	1	1	0
Phosphatidylinositol signaling system	28	0.85525	1	0.5838	0.23374	1	1	0.03053
Porphyrin and chlorophyll metabolism	30	0.91633	1	0.60931	0.21516	1	1	0
Inositol phosphate metabolism	30	0.91633	1	0.60931	0.21516	1	1	0.02867
Primary bile acid biosynthesis	46	1.405	1	0.76521	0.11622	1	1	0.02239

Table S11: Results obtained from Pathway Enrichment Analysis of metabolomic datasets pertaining to ALS A315T mutant TDP-43 Mice Motor Neuron using KEGG DATABASE.

	Total	Expected	Hits	Raw p	#NAME?	Holm adjust	FDR	Impact
Aminoacyl-tRNA biosynthesis	48	1.1768	13	1.34E-11	10.873	1.13E-09	1.13E-09	0
Glyoxylate and dicarboxylate metabolism	32	0.78452	6	8.22E-05	4.0851	0.006824	0.003453	0.21694
Arginine biosynthesis	14	0.34323	4	0.000258	3.5879	0.021181	0.007233	0.2538
Glutathione metabolism	28	0.68645	5	0.000441	3.356	0.035687	0.009252	0.12267
Valine, leucine and isoleucine biosynthesis	8	0.19613	3	0.0007	3.1548	0.056014	0.011763	0
Arginine and proline metabolism	38	0.93161	5	0.00188	2.7258	0.14854	0.026324	0.44097
Phenylalanine, tyrosine and tryptophan biosynthesis	4	0.098065	2	0.003406	2.4678	0.26564	0.040867	1
Alanine, aspartate and glutamate metabolism	28	0.68645	4	0.004125	2.3846	0.31764	0.043314	0.3109
Nitrogen metabolism	6	0.1471	2	0.008253	2.0834	0.62723	0.069325	0
D-Glutamine and D-glutamate metabolism	6	0.1471	2	0.008253	2.0834	0.62723	0.069325	0.5
Citrate cycle (TCA cycle)	20	0.49032	3	0.011624	1.9346	0.8602	0.088768	0.16809
Phenylalanine metabolism	10	0.24516	2	0.023271	1.6332	1	0.1629	0.35714
Glycine, serine and threonine metabolism	33	0.80903	3	0.044701	1.3497	1	0.28884	0.26997
Butanoate metabolism	15	0.36774	2	0.050279	1.2986	1	0.30167	0
Propanoate metabolism	23	0.56387	2	0.10726	0.96955	1	0.60067	0
Galactose metabolism	27	0.66194	2	0.14011	0.85353	1	0.73558	0.03888
Porphyrin and chlorophyll metabolism	30	0.73548	2	0.16602	0.77985	1	0.82032	0
Ascorbate and aldarate metabolism	8	0.19613	1	0.18047	0.74359	1	0.84221	0
Ubiquinone and other terpenoid-quinone biosynthesis	9	0.22065	1	0.20067	0.69752	1	0.88152	0
Biosynthesis of unsaturated fatty acids	36	0.88258	2	0.21996	0.65766	1	0.88152	0
Biotin metabolism	10	0.24516	1	0.22038	0.65683	1	0.88152	0
Valine, leucine and isoleucine degradation	40	0.98065	2	0.25678	0.59044	1	0.98042	0
Tyrosine metabolism	42	1.0297	2	0.27528	0.56023	1	1	0.22844
Primary bile acid biosynthesis	46	1.1277	2	0.31224	0.50552	1	1	0.05823
Fatty acid biosynthesis	47	1.1523	2	0.32144	0.49291	1	1	0.01473
Histidine metabolism	16	0.39226	1	0.32908	0.4827	1	1	0
Glycerolipid metabolism	16	0.39226	1	0.32908	0.4827	1	1	0.09346
Starch and sucrose metabolism	18	0.44129	1	0.36192	0.44139	1	1	0.05023
Pantothenate and CoA biosynthesis	19	0.46581	1	0.37775	0.4228	1	1	0
Pentose phosphate pathway	22	0.53935	1	0.42297	0.37369	1	1	0
Lysine degradation	25	0.6129	1	0.46498	0.33256	1	1	0
Glycolysis / Gluconeogenesis	26	0.63742	1	0.47832	0.32029	1	1	0.00021
Purine metabolism	65	1.5935	2	0.47937	0.31933	1	1	0.00528
Phosphatidylinositol signaling system	28	0.68645	1	0.50402	0.29756	1	1	0.03736
Inositol phosphate metabolism	30	0.73548	1	0.52848	0.27697	1	1	0.12939
Cysteine and methionine metabolism	33	0.80903	1	0.56299	0.2495	1	1	0.10446
Glycerophospholipid metabolism	36	0.88258	1	0.59504	0.22546	1	1	0.01324
Fatty acid elongation	39	0.95613	1	0.62479	0.20427	1	1	0
Fatty acid degradation	39	0.95613	1	0.62479	0.20427	1	1	0
Pyrimidine metabolism	39	0.95613	1	0.62479	0.20427	1	1	0
Tryptophan metabolism	41	1.0052	1	0.64343	0.1915	1	1	0.14305
Steroid biosynthesis	42	1.0297	1	0.65241	0.18548	1	1	0.0282
Steroid hormone biosynthesis	85	2.0839	1	0.88582	0.052656	1	1	0.00528

Table S12: Results obtained from Pathway Enrichment Analysis of metabolomic datasets pertaining to ALS Patient CSF using KEGG DATABASE.

Pathways	Total	Expected	Hits	Raw p	FDR	Impact
Fatty acid degradation	45	1.5222	5	0.015808	0.9539	0.29545
Glycine, serine and threonine metabolism	32	1.0825	4	0.020655	0.9539	0.45161
Glycerolipid metabolism	63	2.1311	5	0.057673	0.9539	0.19355
Glyoxylate and dicarboxylate metabolism	46	1.556	4	0.066387	0.9539	0.26667
Alanine, aspartate and glutamate metabolism	31	1.0486	3	0.084174	0.9539	0.2
Pyruvate metabolism	138	4.6681	8	0.086622	0.7539	0.26277
Sulfur metabolism	37	1.2516	3	0.12661	0.7539	0.38889
Lysine biosynthesis	21	0.71036	2	0.15675	0.7539	0.4
Aminoacyl-t-RNA biosynthesis	41	1.3869	3	0.15843	0.7539	0.4
Cyanoamino acid metabolism	49	1.6575	3	0.22822	0.7539	0.125
Beta-alanine metabolism	73	2.4693	4	0.23061	0.7539	0.19444
Fructose and mannose metabolism	28	0.94715	2	0.2441	0.7539	0.18519
Valine, leucine and isoleucine degradation	51	1.7252	3	0.24657	0.7539	0.36
Valine, leucine and isoleucine biosynthesis	32	1.0825	2	0.29523	0.7539	0.12903
Glutathione metabolism	34	1.1501	2	0.3207	0.7539	0.18182
Methane metabolism	36	1.2178	2	0.34597	0.7539	0.17143
Histidine metabolism	38	1.2854	2	0.37096	0.7539	0.54054
Thiamine metabolism	39	1.3192	2	0.38333	0.7539	0.10526

Table S13: Results obtained from integrated pathway analysis (RNA sequencing and metabolomics) of yeast transformed with TDP-43 and its mutant.

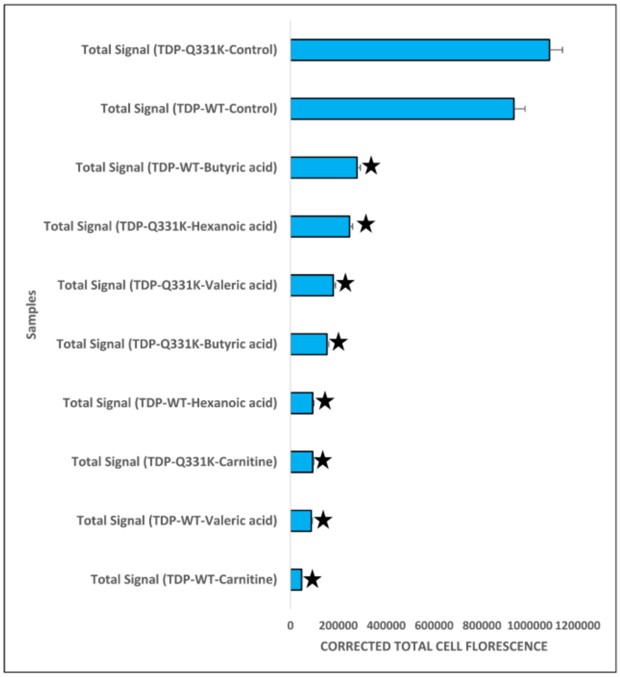

SAMPLES	CTCF_1	CTCF_2	CTCF_3	CTCF_4	T.test	Mean
Total Signal (TDP-WT-Control)	891565.0833	820153.375	1068544	953856.6		933529.8
Total Signal (TDP-Q331K-Control)	991157.8521	892776.425	1108310	1335619		1081966
Total Signal (TDP-WT-Butyric acid)	240216.4167	242642.6667	393567.1	236025.6	5.63449E-05	278112.9
Total Signal (TDP-Q331K-Butyric acid)	156961.25	143111.3333	150896.1	159179.2	6.72232E-05	152537
Total Signal (TDP-WT-Valeric acid)	90716.66667	88522.25	85266.33	83051.83	3.68474E-06	86889.27
Total Signal (TDP-Q331K-Valeric acid)	284426.6667	152353.0833	135702	143482.2	0.000113416	178991
Total Signal (TDP-WT-Hexanoic acid)	101813.5	105317.6667	85137.25	80410.75	3.99237E-06	93169.79
Total Signal (TDP-Q331K-Hexanoic acid)	358425.8333	255500.1667	191006.8	182997.9	0.000195224	246982.7
Total Signal (TDP-WT-Carnitine)	54300.66667	42711.5	44109.25	44759.5	2.81245E-06	46470.23
Total Signal (TDP-Q331K-Carnitine)	96192.83333	99442.83333	87927.58	88155.92	4.70857E-05	92929.79

Table S14: Bar graph and table representing results obtained from fluorescence quantification (Total Cell fluorescence) of for TDP-43 transformed yeast cells treated with short chain fattyacids. [P-VALUE<0.05 ★]

SAMPLES	Ratio_1	Ratio_2	Ratio_3	Ratio_4	Mean
Total Signal (TDP-WT-Control)	1.014612089	1.035257843	1.050863	1.152909	1.06341
Total Signal (TDP-Q331K-Control)	1.034097271	1.053355261	1.241007	1.266407	1.148717
Total Signal (TDP-WT-Butyric acid)	1	1.011324277	1	1	1.002831
Total Signal (TDP-Q331K-Butyric acid)	1	1	1	1	1
Total Signal (TDP-WT-Valeric acid)	1	1	1	1	1
Total Signal (TDP-Q331K-Valeric acid)	1	1	1	1	1
Total Signal (TDP-WT-Hexanoic acid)	1	1	1	1	1
Total Signal (TDP-Q331K-Hexanoic acid)	1	1	1	1	1
Total Signal (TDP-WT-Carnitine)	1	1	1	1	1
Total Signal (TDP-Q331K-Carnitine)	1	1	1	1	1

Table S15: Table representing results of ratios obtained between Total cell fluorescence and aggregate fluorescence for TDP-43 transformed yeast cells treated with short chain fattyacids.

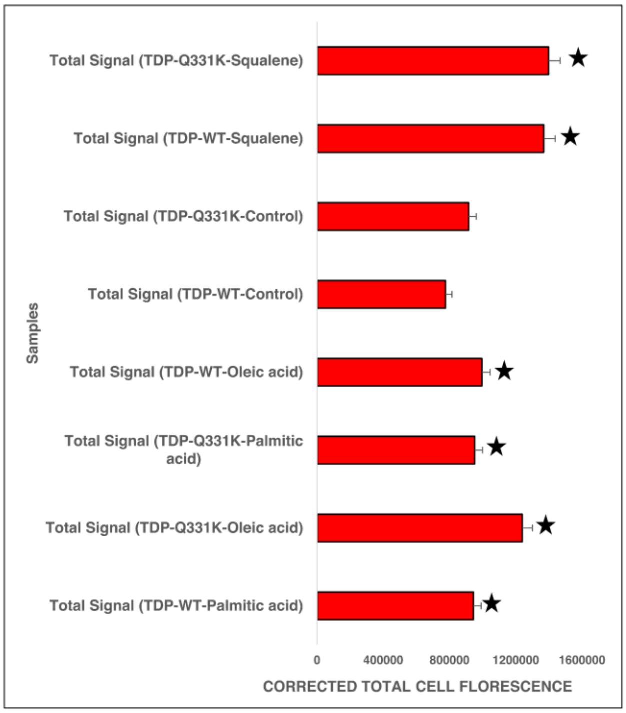

SAMPLES	CTCF_1	CTCF_2	CTCF_3	CTCF_4	T.test	Mean
Total Signal (TDP-WT-Control)	8,91,565.08	820153.375	1068544	953856.6		933529.8
Total Signal (TDP-Q331K-Control)	10,62,307.25	1061798.5	1108310	1335619		1142009
Total Signal (TDP-WT-Palmitic acid)	14,79,771.83	1440714.833	1334150	1433519	0.0002041	1422039
Total Signal (TDP-Q331K-Palmitic acid)	15,21,101.08	1481171.5	1557093	1476803	0.001682246	1509042
Total Signal (TDP-WT-Oleic acid)	11,46,026.00	1207783.833	1196998	1151576	0.004533494	1175596
Total Signal (TDP-Q331K-Oleic acid)	12,55,279.58	1316697.5	1311766	1467343	12,55,279.58	1316697.5
Total Signal (TDP-WT-Squalene)	16,19,724.67	1669217.917	2363144	2155367	0.001737097	1951863
Total Signal (TDP-Q331K-Squalene)	16,74,394.67	1687129.378	2339941	2215607	0.004109394	1979268

Table S16: Bar graph and table representing results obtained from fluorescence quantification (Total Cell fluorescence) of for TDP-43 transformed yeast cells treated with long chain fattyacids. [P-VALUE<0.05★]

SAMPLES	Ratio_1	Ratio_2	Ratio_3	Ratio_4	Mean
Total Signal (TDP-WT-Control)	1.013614067	1.035257843	1.050863	1.122216	1.055487772
Total Signal (TDP-Q331K-Control)	1.108329037	1.108155463	1.241007	1.238492	1.173995743
Total Signal (TDP-WT-Palmitic acid)	1.309945576	1.055929819	1.054309	1.120912	1.135274053
Total Signal (TDP-Q331K-Palmitic acid)	1.427402014	1.130670518	1.185807	1.217045	1.24023116
Total Signal (TDP-WT-Oleic acid)	1.028540606	1.015492252	1.058989	1.053673	1.039173551
Total Signal (TDP-Q331K-Oleic acid)	1.004076382	1.006193332	1.110198	1.017824	1.034572979
Total Signal (TDP-WT-Squalene)	1.078263745	1.083709099	1.198555	1.202553	1.140770349
Total Signal (TDP-Q331K-Squalene)	1.190447148	1.284831157	1.136518	1.120141	1.182984267

Table S17: Table representing results of ratios obtained between Total cell fluorescence and aggregate fluorescence for TDP-43 transformed yeast cells treated with long chain fattyacids.

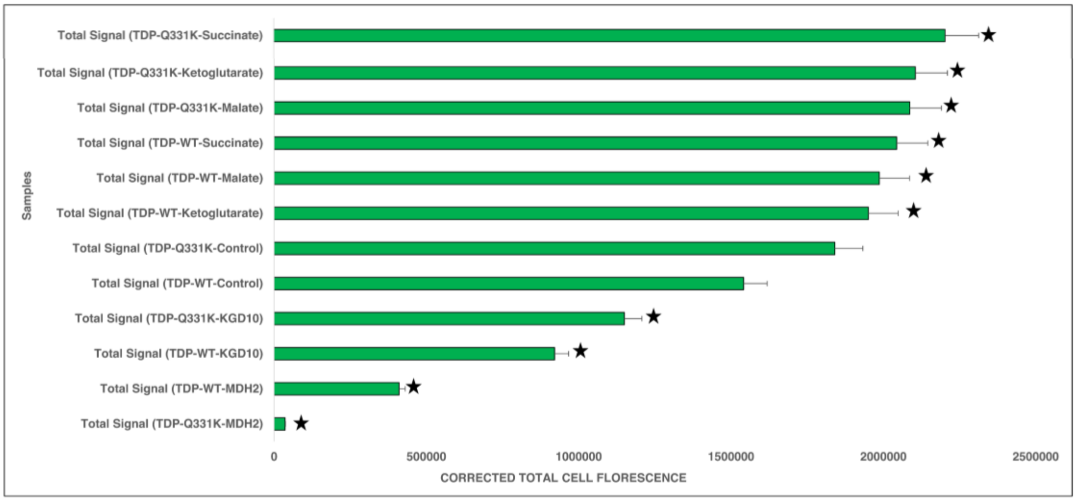

Table S18: Bar graph representing results obtained from fluorescence quantification (Total Cell fluorescence) of for TDP-43 transformed yeast cells treated with TCA cycle metabolites and knock-outs. [P-VALUE<0.05 ★]

SAMPLES	CTCF_1	CTCF_2	CTCF_3	CTCF_4	T.test	Mean
Total Signal (TDP-WT-Control)	14,35,512.58	15,05,059.56	15,82,521.00	16,41,610.00		1541175.785
Total Signal (TDP-Q331K-Control)	17,77,243.92	19,18,254.08	17,72,910.58	18,93,865.08		1840568.417
Total Signal (TDP-WT-Citrate)	20,67,983.42	21,52,615.08	21,57,146.58	22,35,757.25	3.6734E-05	2153375.583
Total Signal (TDP-Q331K-Citrate)	19,00,663.08	23,38,854.42	22,27,943.75	22,71,074.00	0.016611891	2184633.813
Total Signal (TDP-WT-Ketoglutarate)	17,82,512.58	20,77,940.08	19,32,205.00	20,09,812.42	0.001892746	1950617.521
Total Signal (TDP-Q331K-Ketoglutarate)	18,00,222.92	22,73,555.25	21,79,163.75	21,66,871.42	0.054774127	2104953.333
Total Signal (TDP-WT-Succinate)	18,60,447.25	21,38,521.08	19,75,440.42	21,99,872.08	0.001355415	2043570.208
Total Signal (TDP-Q331K-Succinate)	19,24,524.33	23,43,181.75	23,79,615.08	21,65,094.25	0.016985664	2203103.854
Total Signal (TDP-WT-Malate)	16,16,355.22	19,90,769.25	21,72,199.42	21,66,565.75	0.261879837	1986472.41
Total Signal (TDP-Q331K-Malate)	17,76,731.06	20,32,291.75	22,80,681.25	22,55,936.92	0.543343782	2086410.243
Total Signal (TDP-WT-MDH2)	5,09,289.89	3,70,572.25	3,48,025.08	4,12,905.58	7.25477E-07	410198.2014
Total Signal (TDP-Q331K-MDH2)	13,137.30	40,837.83	38,337.83	51,671.17	1.08622E-06	35996.03333
Total Signal (TDP-WT-KGD10)	9,84,229.45	8,40,313.61	9,06,912.17	9,50,912.50	1.03093E-05	920591.9313
Total Signal (TDP-Q331K-KGD10)	10,98,639.38	11,06,139.38	11,13,639.38	12,80,306.04	8.54437E-05	1149681.042

Table S18: Table representing results obtained from fluorescence quantification (Total Cell fluorescence) of for TDP-43 transformed yeast cells treated with TCA cycle metabolites and knock-outs.

SAMPLES	Ratio_1	Ratio_2	Ratio_3	Ratio_4	Mean
Total Signal (TDP-WT-Control)	1.125256342	1.167536193	1.27183928	1.151524993	1.179039202
Total Signal (TDP-Q331K-Control)	1.258497677	1.243388702	1.205485804	1.28523048	1.248150666
Total Signal (TDP-WT-Ketoglutarate)	1.08266107	1.045840824	1.106749415	1.011551724	1.061700758
Total Signal (TDP-Q331K-Ketoglutarate)	1.10947612	1.131197381	1.073414508	1.036871081	1.087739773
Total Signal (TDP-WT-Succinate)	1.148093325	1.066051593	1.080601756	1.144155606	1.10972557
Total Signal (TDP-Q331K-Succinate)	1.151482561	1.041375272	1.089708674	1.033636933	1.07905086
Total Signal (TDP-WT-Malate)	1.127883725	1.198904631	1.176186849	1.172044385	1.168754897
Total Signal (TDP-Q331K-Malate)	1.061034846	1.115907289	1.122368558	1.059755237	1.089766483
Total Signal (TDP-WT-MDH2)	1	1	1	1	1
Total Signal (TDP-Q331K-MDH2)	1	1	1	1	1
Total Signal (TDP-WT-KGD10)	1.197314074	1.021919525	1.18322226	1.179257961	1.145428455
Total Signal (TDP-Q331K-KGD10)	1.144319017	1.14151664	1.083050287	1.104872351	1.118439574

Table S19: Table representing results obtained from fluorescence quantification (Total Cell fluorescence) of for TDP-43 transformed yeast cells treated with TCA cycle metabolites and knock-outs.

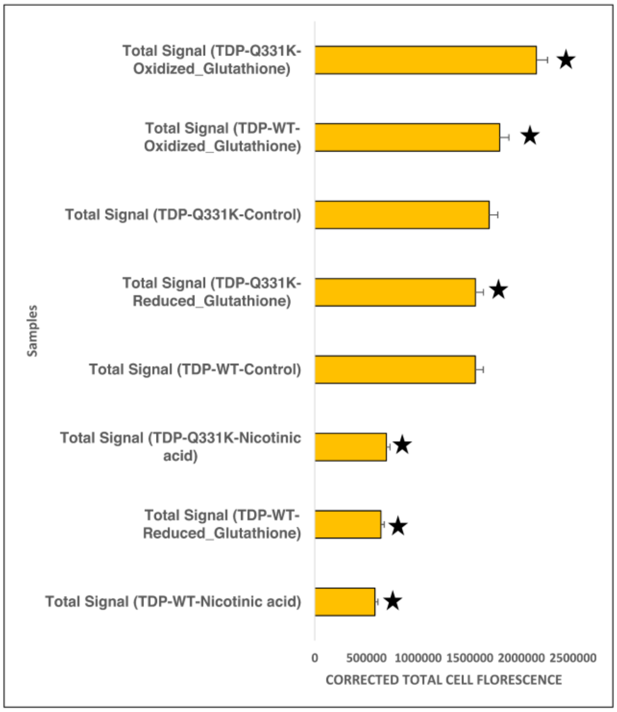

SAMPLES	CTCF_1	CTCF_2	CTCF_3	CTCF_4	T.test	Mean
Total Signal (TDP-WT-Control)	16,50,152.58	15,77,285.58	15,67,866.42	14,17,776.92		1553270.375
Total Signal (TDP-Q331K-Control)	17,91,279.08	16,85,129.25	16,80,905.75	15,91,712.25		1687256.583
Total Signal (TDP-WT-Oxidized Glutathione)	18,38,306.42	17,51,542.58	18,41,593.08	17,24,965.58	0.006186002	1789101.917
Total Signal (TDP-Q331K-Oxidized Glutathione)	20,97,257.92	21,05,804.25	22,10,698.83	21,66,553.92	8.33639E-05	2145078.729
Total Signal (TDP-WT-Reduced Glutathione)	6,39,752.25	7,73,549.08	5,02,861.67	6,43,574.33	1.68574E-05	639934.3333
Total Signal (TDP-Q331K-Reduced Glutathione)	14,05,881.75	17,95,222.92	12,19,880.83	17,95,222.92	0.408665775	1554052.104
Total Signal (TDP-WT-Nicotinic acid)	5,85,784.50	6,28,070.17	6,28,572.00	4,84,486.67	3.32822E-06	581728.3333
Total Signal (TDP-Q331K-Nicotinic acid)	8,47,220.17	6,44,370.17	7,06,276.33	5,74,361.50	8.23355E-06	693057.0417

Table S20: Table representing results of ratios obtained between Total cell fluorescence and aggregate fluorescence for TDP-43 transformed yeast cells treated glutathione, nicotinic acid and knock-outs. [P-VALUE<0.05 ★]

SAMPLES	Ratio_1	Ratio_2	Ratio_3	Ratio_4	Mean
Total Signal (TDP-WT-Control)	1.191878341	1.157431112	1.149505443	1.056194791	1.138752422
Total Signal (TDP-Q331K-Control)	1.060769431	1.212780975	1.147278831	1.177272935	1.149525543
Total Signal (TDP-WT-Oxidized_Glutathione)	1.072818761	1.034412499	1.126465024	1.007984477	1.06042019
Total Signal (TDP-Q331K-Oxidized_Glutathione)	1.085488718	1.056263684	1.176735904	1.127471394	1.111489925
Total Signal (TDP-WT-Reduced_Glutathione)	1.302026079	1.266422957	1.108340518	1.546499152	1.305822177
Total Signal (TDP-Q331K-Reduced_Glutathione)	1.115799018	1.108774915	1.072341794	1.108774915	1.101422661
Total Signal (TDP-WT-Nicotinic acid)	1.916650125	2.348635569	1.092895788	2.704048743	2.015557556
Total Signal (TDP-Q331K-Nicotinic acid)	2.27828301	1.702434086	1.355644517	2.324496946	1.91521464

Table S21: Bar graph and table representing results obtained from fluorescence quantification (Total Cell fluorescence) for TDP-43 transformed yeast cells treated with glutathione and nicotinic acid.

Treatments	Fold Change	Treatments	Fold Change
Total Protein-TDP-WT Palmitic acid	1.253545097	Total Protein-TDP-Q331K Palmitic acid	1.588137868
Total Protein-TDP-WT Butyric acid	0.058266114	Total Protein-TDP-Q331K Butyric acid	0.315693773
Soluble Protein-TDP-WT Palmitic acid	1.626951431	Soluble Protein-TDP-Q331K Palmitic acid	1.186988761
Soluble Protein-TDP-WT Butyric acid	0.118795761	Soluble Protein-TDP-Q331K Butyric acid	0.264276471
Insoluble Protein-TDP-WT Palmitic acid	1.204168565	Insoluble Protein-TDP-Q331K Palmitic acid	1.389558969
Insoluble Protein-TDP-WT Butyric acid	0.068800013	Insoluble Protein-TDP-Q331K Butyric acid	0.291366691

Table S22 : Quantification results of filter retardation assay carried out on treated Saccharomyces cerevisiae transformed with TDP-43 and its mutant.

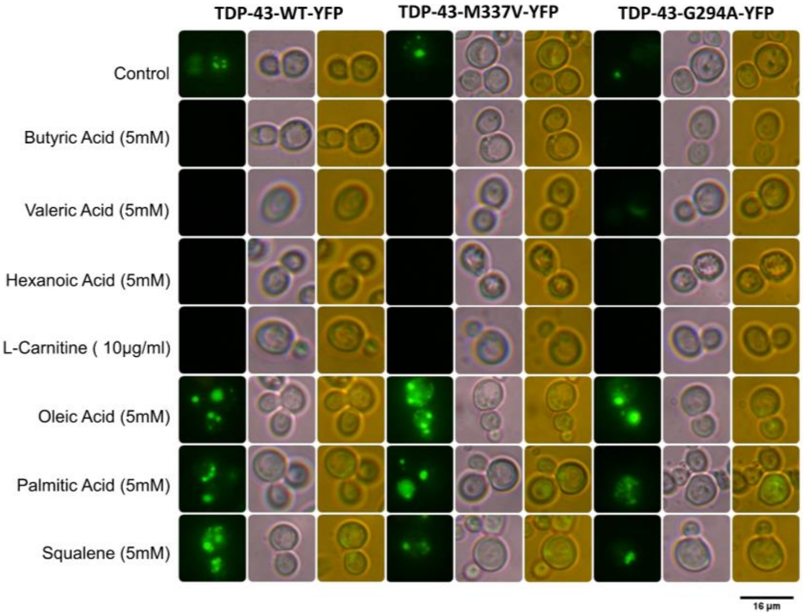

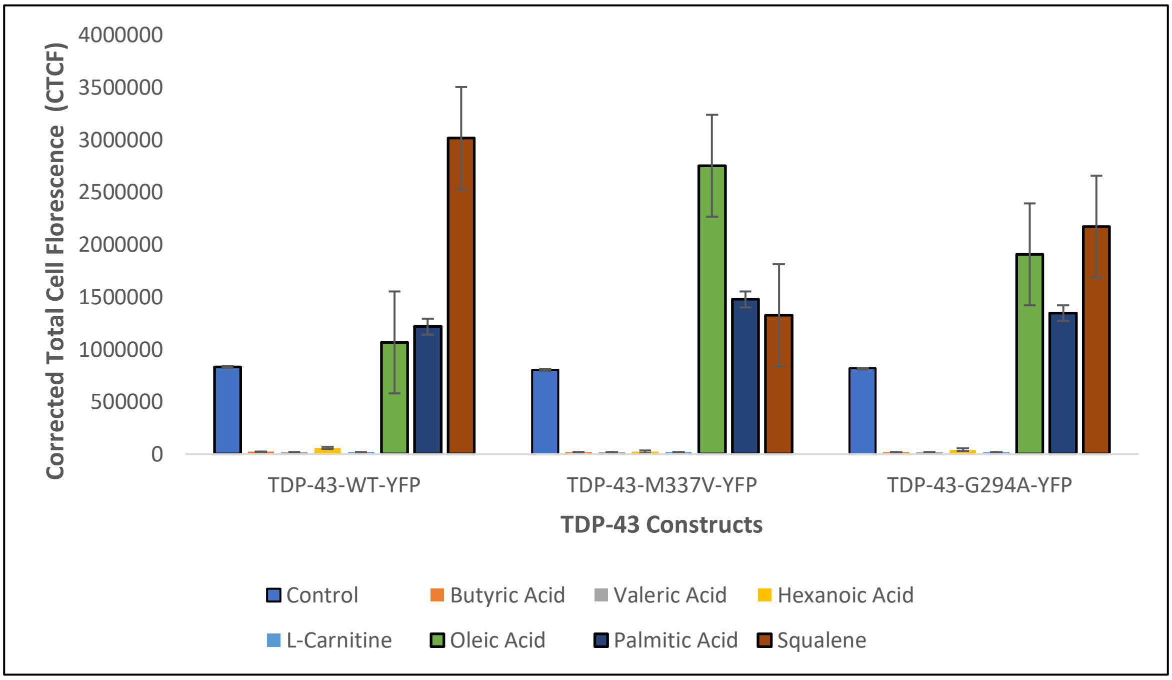

T.Test (P.Value)	TDP-43-WT-YFP	TDP-43-M337V-YFP	TDP-43-G294A-YFP
Control	2.44011E-06	0.000427338	4.2448E-08
Butyric Acid	2.40272E-06	0.000432605	0.218022605
Valeric Acid	2.40272E-06	0.000432605	0.218022605
Hexanoic Acid	3.52257E-06	0.00044243	4.93032E-08
L-Carnitine	2.3402E-06	0.000426621	1.36252E-08
Oleic Acid	0.120670489	1.84404E-05	9.45731E-10
Palmitic Acid	0.007426258	0.018898981	0.002811746
Squalene	1.79727E-06	0.03036374	0.00098102

Table S23 : Imaging (dark field, bright field and overlay) and quantification results of metabolite addition experiments show that short chain fattyacids reduces amyloidogenesis while long chain fattyacids increase amyloidogenesis in Saccharomyces cerevisiae transformed with TDP-43 and its mutant.

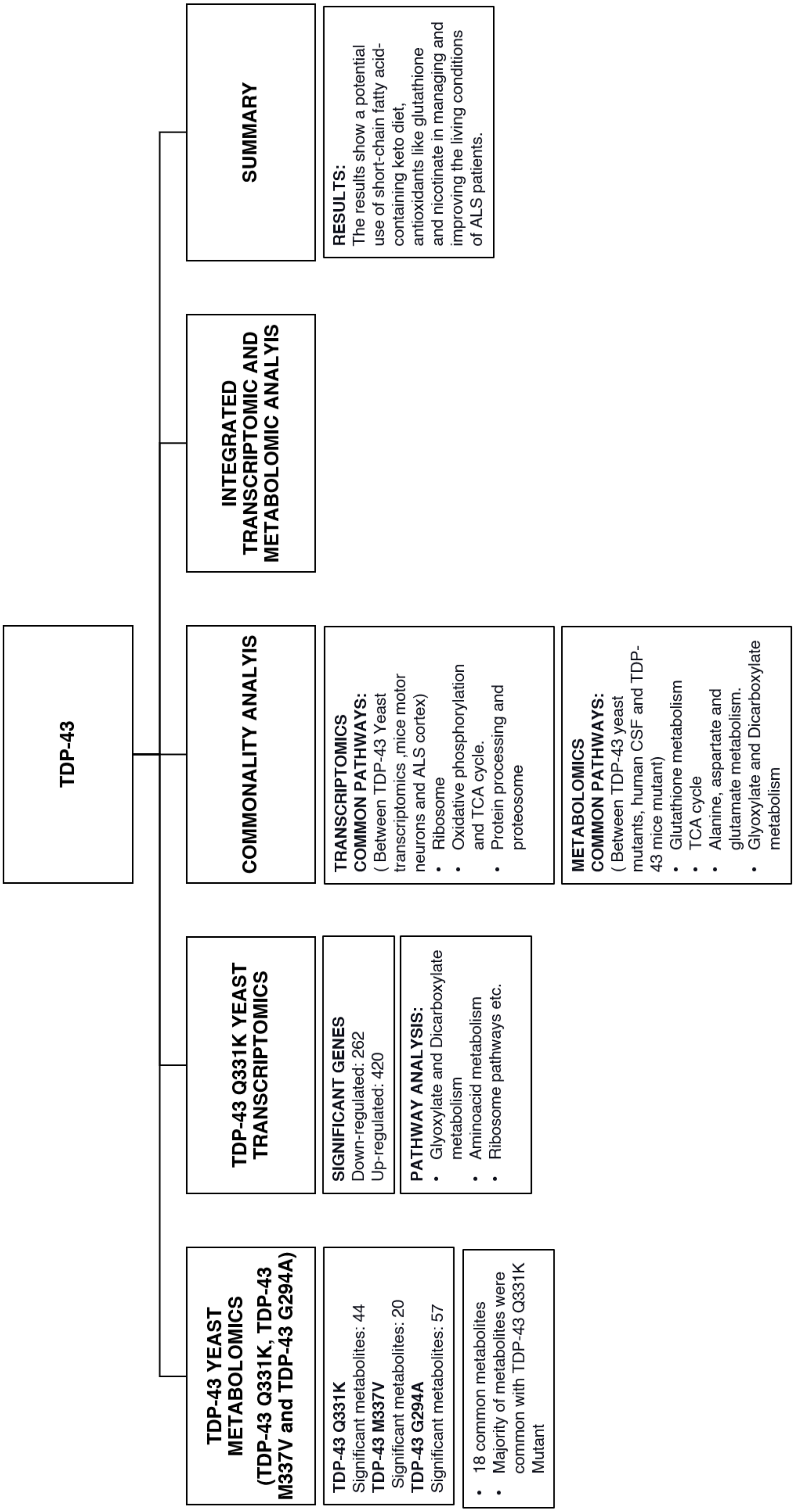

Table S24: Figure summarizing the findings of the study.
